# Predicting the prognosis of breast cancer patients by using nutrition-based index: a systematic review and meta-analysis

**DOI:** 10.3389/fonc.2026.1775719

**Published:** 2026-05-11

**Authors:** Si Chen, Jun Miao, Xin Cheng, Yan Duan

**Affiliations:** 1 The Clinical Medical Research Center of Breast and Thyroid Tumor in Xinjiang, Tumor Hospital Affiliated to Xinjiang Medical University, Urumqi, China; 2 Medical Imaging Center, Cancer Hospital Affiliated to Xinjiang Medical University, Urumqi, China

**Keywords:** breast cancer, controlling nutritional status, meta-analysis, prognosis, prognostic nutritional index

## Abstract

**Background:**

The prognostic nutritional index (PNI) and the controlling nutritional status (CONUT) score are composite markers of nutritional and inflammatory status that are used to predict tumor progression. Their prognostic relevance in breast cancer (BC) has been reported, but the evidence remains inconsistent. Therefore, the prognostic utility of PNI and CONUT in BC was evaluated in this meta-analysis.

**Methods:**

A search was carried out across four major databases: PubMed, Embase, Web of Science, and the Cochrane Library, from inception to 20 October 2025. Studies assessing the association of PNI or CONUT with clinical outcomes in BC were included based on prespecified eligibility criteria. Overall survival (OS), disease-free survival (DFS), and pathological complete response (pCR) were analyzed, and pooled effects were reported as hazard ratios (HRs) or odds ratios (ORs) with 95% confidence intervals (CIs). Subgroup analyses, meta-regression, and 95% prediction intervals (PIs) were used to explore heterogeneity.

**Results:**

A total of 32 reports comprising 13,120 individuals with BC were included. Higher PNI was linked with improved OS (HR = 0.49, 95% CI 0.39-0.61; P < 0.00001; I² = 83%) and longer DFS (HR = 0.69, 95% CI 0.58-0.81; P < 0.00001; I² = 83%), whereas the evidence for an association between PNI and pCR was inconclusive (OR = 1.57, 95% CI 1.00-2.47; P = 0.05; I² = 83%), and the result was sensitive to individual studies. In addition, higher CONUT corresponded to poorer OS (HR = 1.77, 95% CI 1.29-2.44; P = 0.0005; I² = 77%) and shorter DFS (HR = 2.08, 95% CI 1.75-2.47; P < 0.00001; I² = 6%). Prediction intervals for PNI and OS, PNI and DFS, PNI and pCR, and CONUT and OS all crossed the null value, indicating substantial between-study variability in the expected effect across future settings.

**Conclusions:**

Across the included studies, higher PNI was associated with better OS and DFS, while elevated CONUT was associated with poorer OS and DFS. Therefore, PNI and CONUT may be considered candidate prognostic indicators in BC.

Systematic review registration: PROSPERO, identifier CRD420251249070.

## 1 Introduction

Breast cancer is the leading malignancy among women, with 2.308 million new cases diagnosed globally in 2022. With respect to mortality, this malignancy ranks as the second leading cause of cancer-associated deaths in the female population, following lung cancer (1). Breast cancer is characterized by marked heterogeneity, and prognosis is determined by tumor size, histologic grade, lymph node status, and molecular biomarkers, including hormone receptor status, human epidermal growth factor receptor 2 (HER2) status, and BRCA1 alterations. Although gene expression-based assays are used for prognostic stratification, their high-cost limits routine adoption in most clinical settings (2, 3).

Systemic inflammatory activation is implicated in malignant progression (4). Inflammatory serum biomarkers and composite scores have been validated as prognostic indicators across multiple cancers (5, 6), including the neutrophil-to-lymphocyte ratio (NLR), platelet-to-lymphocyte ratio (PLR), and lymphocyte-to-monocyte ratio (LMR). Nutritional status is also regarded as a key and potentially modifiable contributor to cancer development (7). In individuals with cancer, malnutrition is linked to reduced responsiveness to anticancer therapy, increased postoperative complication risk, and worse OS (8). Collectively, inflammatory and nutritional biomarkers are considered prognostic factors in breast cancer.

PNI is a nutrition- and inflammation-related biomarker, computed from serum albumin levels and the count of peripheral blood lymphocytes (9). PNI is reported to be associated with prognosis and treatment response across multiple malignancies (10). As a cost-effective and readily available blood-based marker, an elevated PNI is typically linked to better clinical prognosis in individuals with BC (11). In a single-center study of 60 patients with metastatic BC undergoing chemotherapy, higher pretreatment PNI is reported to predict improved OS and longer DFS after 1 year of follow-up (12). The CONUT score is another blood-based index derived from total cholesterol, serum albumin, and lymphocyte count (13). CONUT is also linked to oncologic outcomes in several cancers, and higher CONUT scores are typically associated with poorer prognosis in BC. In a retrospective Chinese cohort of 1,367 surgically treated patients, higher preoperative CONUT is reported to predict lower OS and shorter DFS (14). Despite these observations, the predictive utility of PNI and CONUT in BC remains to be further clarified.

Although a prior meta-analysis supports the clinical utility of PNI and CONUT for OS, DFS, and pCR in breast cancer (15), only studies published before 2023 are included. Since then, numerous additional clinical studies have been published, and the findings are not fully consistent. Accordingly, 15 studies published between 2023 and 2025 are additionally included in the present meta-analysis (7). Because consensus has not been reached across these newer reports, the prognostic utility of PNI and CONUT in BC is systematically re-evaluated by integrating the most recent evidence with previously available data.

## 2 Materials and methods

### 2.1 Literature search

The present systematic review adhered to the Preferred Reporting Items for Systematic Reviews and Meta-Analyses (PRISMA 2020) guideline (16). The protocol was registered in the International Prospective Register of Systematic Reviews (PROSPERO: CRD420251249070). A search strategy was developed by two investigators (SC and YD). Subject terms and keywords were independently generated and used to search PubMed, Embase, Web of Science, and the Cochrane Library from database inception to 20 October 2025. Search terms included “Prognostic Nutritional Index,” “PNI,” “CONUT,” “CONUT score,” “Controlling nutritional status,” “Breast Neoplasm,” “Breast Tumor,” “Breast Cancer,” “Breast Malignant Neoplasm,” and “Mammary Cancers.” The full search strategy was provided in **Supplementary Table 1**.

### 2.2 Study selection

Eligibility required that studies fulfill all of the following requirements: (1) human participants were enrolled and breast cancer was confirmed by histopathology; (2) the prognostic association of PNI or CONUT with OS, DFS or pCR was evaluated; (3) effect size measures, namely HRs and ORs, together with their 95% CIs, were either explicitly reported or could be calculated from the available data. (4) patients were stratified into high versus low PNI or CONUT groups using a prespecified cutoff value; and (5) the study was published in full. Studies were excluded if they (1) were reviews, commentaries, conference abstracts, case reports, or letters; (2) provided insufficient information to estimate HRs/ORs with 95% CIs; (3) did not report survival or response outcomes; or (4) contained duplicate or overlapping datasets. Two investigators (SC and YD) independently reviewed titles and abstracts, obtained full texts, and assessed eligibility. Discrepancies were resolved by consensus.

### 2.3 Data extraction

From each eligible report, study-level details and outcome metrics were compiled by a pair of reviewers (SC and YD) working in parallel. When discordant entries arose, a unified decision was reached by the full author team. The dataset included the first author’s name, year of publication, study location, study design, sample size, patient age, study population, study duration, treatment regimen, follow-up time, tumor stage, PNI cutoff value, CONUT cutoff value, time of assessment, and the corresponding HRs or ORs with 95% CIs for OS, DFS, and pCR. To minimize confounding, multivariable-adjusted HRs were preferentially extracted; otherwise, univariable HRs or Kaplan–Meier estimates were used.

### 2.4 Quality assessment

Methodological rigor was appraised with the Newcastle–Ottawa Scale (NOS) for all eligible observational studies. Ratings were assigned in three categories: participant selection, between-group comparability, and outcome assessment, yielding a possible maximum of 9 points (17, 18).

### 2.5 Statistical analysis

All computations were performed using STATA 15.0 and Review Manager 5.4, with a two-sided α level of 0.05. For each eligible report, effect measures for OS, DFS, and pCR were extracted as HRs or ORs with 95% CIs. Between-study heterogeneity was assessed using Cochran’s Q test and the Higgins I² statistic (19); I² > 50% or P < 0.05 was considered to indicate substantial heterogeneity. A fixed-effect model was used when heterogeneity was negligible, whereas a random-effects model was adopted when heterogeneity was evident. Subgroup analyses and meta-regression were conducted to explore potential sources of heterogeneity. In meta-regression analyses, residual heterogeneity (τ²) and explained variance (pseudo-R²) were additionally recorded to quantify unexplained and explained between-study variability, respectively. For pooled analyses performed under random-effects models, 95% prediction intervals (PIs) were also calculated to estimate the range of effects expected in a future similar study. Sensitivity analyses were conducted by sequentially omitting individual studies. Publication bias was assessed using funnel plots and the Egger and Begg tests, and when appropriate, the trim-and-fill method was applied to examine the robustness of the pooled estimates.

## 3 Results

### 3.1 Study characteristics

**Figure 1:**
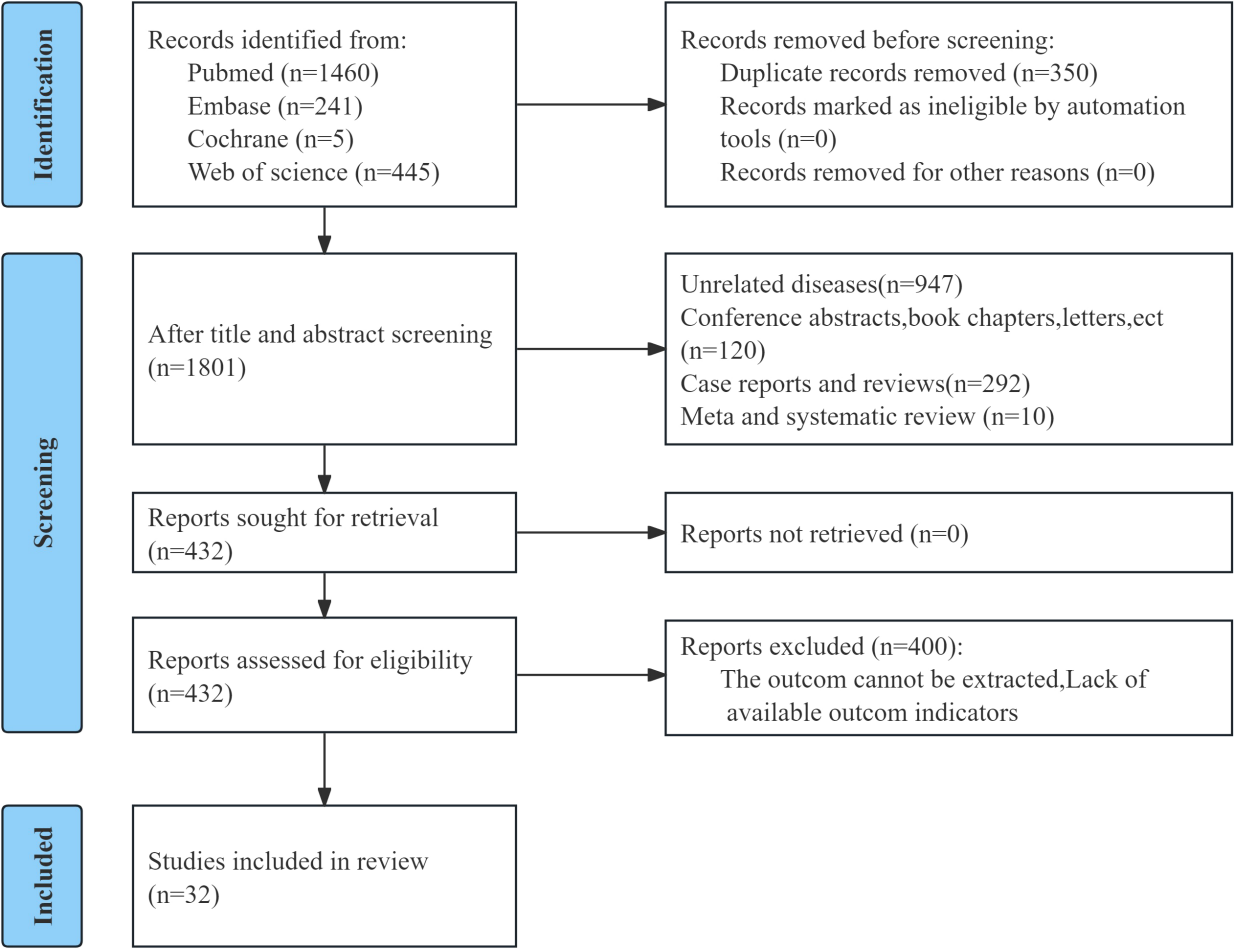
Flow chart of literature screening.

A total of 2,151 citations were retrieved at the first search step; after deduplication, 350 repeated items were eliminated. After title and abstract screening, 1,369 records were excluded. Full texts of 432 articles were assessed for eligibility, and 400 were excluded, primarily because data required for survival analyses were insufficient. Ultimately, 32 studies comprising 13,120 patients were included in the meta-analysis (**Figure 1**), with sample sizes spanning from 35 to 1,367.

Across the 32 included studies published between 2014 and 2025, 37 comparison groups were extracted. Of these, 26 groups were conducted in Asia (11, 12, 14, 18, 20–38), 6 in non-Asian settings (17, 39–42), and 5 were multicenter cohorts (7, 43–45). Most groups were retrospective, and two were prospective (45). All groups were published in English, with study periods spanning 1998 to 2025. The median patient age ranged from 47 to 66 years. PNI-based outcomes were reported in 30 groups, whereas CONUT-based outcomes were reported in 7 groups. Regarding PNI measurement, pretreatment PNI was extracted in 27 comparison groups, whereas the timing of assessment was not reported in the remaining 3 comparison groups. CONUT was measured before treatment in 6 groups, and the timing was not reported in 1 group. For studies yielding multiple comparison groups (designated as a and b), dataset non-overlap was cross-checked and is summarized in **Supplementary Table 3**. Specifically, Hu 2025a and Hu 2025b (22) and Zhang 2023a and Zhang 2023b (45) represented the same underlying cohorts but reported different nutritional indices (PNI vs CONUT), Li 2023a and Li 2023b (27) involved independent patient cohorts defined by different biologic subtypes, and Wang 2025a/2025b (33) and Yildirim 2024a/2024b (42) represented the same underlying cohorts but reported different endpoints (**Supplementary Table 3**). In pooled analyses of a single endpoint, each study was included only once to ensure data independence. In addition, because the majority of included studies originated from China, a separate cross-reference table of Chinese cohorts was constructed to assess potential overlap based on center/hospital, study period, population/subtype, nutritional index, and treatment setting (**Supplementary Table 4**). The HR values for the CONUT score were all adjusted using multivariate regression analysis. For PNI, the values were adjusted in most studies using multivariate regression analysis, whereas univariate analysis results were provided for OS in 5 studies (11, 12, 17, 30, 39), for DFS in 5 studies (11, 24, 28, 29, 39), and for pCR in 1 study (32). For PNI, associations with OS were evaluated in 19 groups, with DFS in 16 groups, and with pCR in 7 groups. For CONUT, associations with OS and DFS were each evaluated in 6 groups. Cut-off values applied to define high versus low PNl varied across studies, spanning 40 to 55, whereas a CONUT score of 3 was used in most studies to categorize high versus low CONUT. Baseline features of the eligible cohorts are presented in **Table 1**.

**Table 1:** Basic characteristics of the included literature.

Author	Study period	Region	Study design	Population	Treatment method	Timing of detection	No. of patients	Median follow- up (months)	Mean/median Age	TNM stage	Cut-off	Outcomes
Amitani, M 2025 (17)	2011-2022	Turkey	Retrospective cohort	MBC	Chemotherapy	Pre-treatment	67	13.8	58.9	IV	48.3(PNI)	OS
Arici, M 2024 (39)	2015-2023	Turkey	Retrospective cohort	BC	NACT and surgery	Pre-treatment	304	38.5	50.0	II-III	54.1(PNI)	OS/DFS
Birsin, Z 2025 (43)	2010-2025	Multicenter	Retrospective cohort	HER2 positive	NACT and surgery	Pre-treatment	174	NA	51.0	II-III	55.0(PNI)	pCR
Buyuksimsek, M 2020 (40)	2006-2019	Turkey	Retrospective cohort	BC	NACT and surgery	Pre-treatment	110	NA	52.0	II-III	50.0(PNI)	pCR
Chen, L 2021 (11)	1998-2016	China	Retrospective cohort	BC	NACT and surgery	Pre-treatment	477	55.0	47.0	I-III	51.0(PNI)	OS/DFS
Gu, H 2025 (20)	2012-2023	China	Retrospective cohort	TNBC	Surgery	Pre-treatment	130	53.0	55.0	NA	51.0(PNI)	OS/DFS
Guo, X 2024 (21)	2010-2021	China	Retrospective cohort	BC	NACT and surgery	Pre-treatment	237	NA	50.0	II-III	47.0(PNI)	pCR
Hu, J 2025a (22)	2012-2018	China	Retrospective cohort	BC	Surgery	Pre-treatment	536	90.0	52.0	I-III	50.2(PNI)	DFS
Hu, J 2025b (22)	2012-2018	China	Retrospective cohort	BC	Surgery	Pre-treatment	536	90.0	52.0	I-III	1.0(CONUT)	DFS
Hua, X 2020 (23)	2010-2012	China	Retrospective cohort	BC	Surgery	Pre-treatment	380	63.1	47.0	I-IV	52.0(PNI)	OS
Huang, Z 2020 (14)	2010-2019	China	Retrospective cohort	BC	Surgery	Pre-treatment	1367	70.3	48.0	I-IV	3.0(CONUT)	OS/DFS
Hutajulu, S 2025 (24)	2018-2024	Indonesia	Retrospective cohort	BC	Chemotherapy	Pre-treatment	202	36.0	51.0	I-IV	54.1(PNI)	OS/DFS
Li, W 2020 (25)	2007-2010	China	Retrospective cohort	BC	Surgery	Pre-treatment	861	61.7	55.0	NA	3.0(CONUT)	OS/DFS
Li, X 2025 (26)	2022-2024	China	Retrospective cohort	Advanced breast cancer	Immunotherapy	Pre-treatment	154	12.0	52.0	IV	47.5(PNI)	OS/DFS
Li, Y 2023a (27)	2014-2017	China	Retrospective cohort	HER2-low BC	Surgery	Pre-treatment	697	70.0	52.0	I-III	3.0(CONUT)	OS/DFS
Li, Y 2023b (27)	2014-2017	China	Retrospective cohort	HER2-positive BC	Surgery	Pre-treatment	202	72.0	55.0	I-III	3.0(CONUT)	OS/DFS
Mohri, T 2016 (28)	2006-2015	Japan	Retrospective cohort	BC	Surgery	Pre-treatment	212	47.7	66.0	I-III	52.8(PNI)	OS/DFS
Oba, T 2020 (29)	2005-2016	Japan	Retrospective cohort	BC	NACT and surgery	NA	191	51.0	51.2	I-III	NA(PNI)	DFS
Oba, T 2021 (12)	2011-2018	Japan	Retrospective cohort	MBC	Chemotherapy	Pre-treatment	60	11.6	58.6	IV	48.3(PNI)	OS/DFS
Onder, T 2025 (41)	2017-2023	Turkey	Retrospective cohort	MBC	CDK4/6i and ET	Pre-treatment	431	35.7	56.0	IV	47.5(PNI)	OS/DFS
Qiu, Y 2024 (30)	2016-2020	China	Retrospective cohort	TNBC	Surgery	Pre-treatment	223	62.0	50.0	I-IV	50.9(PNI)	OS
Qu, F 2023 (44)	2012-2022	Multicenter	Retrospective cohort	BC	NACT and surgery	Pre-treatment	1170	NA	49.0	I-IV	53.0(PNI)	pCR
Shi, J 2023 (7)	2016-2021	Multicenter	Retrospective cohort	BC	NA	NA	776	NA	52.0	I-IV	42.0(PNI)	OS
Sun, L 2023 (31)	2013-2020	China	Retrospective cohort	BC	Surgery	Pre-treatment	135	NA	53.0	NA	51.1(PNI)	DFS
Wang, S 2025 (32)	2018-2023	China	Retrospective cohort	TNBC	NACT and surgery	Pre-treatment	431	NA	48.0	I-IV	53.6(PNI)	pCR
Wang, Y 2019 (18)	2013-2018	China	Retrospective cohort	Locally advanced BC	NACT and surgery	Pre-treatment	202	26.0	NA	III	55.0(PNI)	pCR
Wang, Y 2025a (32)	2017-2022	China	Retrospective cohort	BC	Surgery	Pre-treatment	200	46.0	55.5	I-III	43.6(PNI)	OS
Wang, Y 2025b (33)	2017-2022	China	Retrospective cohort	BC	Surgery	Pre-treatment	200	46.0	55.5	I-III	45.2(PNI)	DFS
Xu, T 2022 (34)	2013-2020	China	Retrospective cohort	BC	Surgery	Pre-treatment	508	49.0	49.0	I-IV	53.0(PNI)	OS/DFS
Yamamoto, S 2022 (35)	2012-2021	Japan	Retrospective cohort	MBC	Chemotherapy	Pre-treatment	110	12.1	50.0	IV	40.0(PNI)	OS
Yamanouchi, K 2023 (36)	2012-2021	Japan	Retrospective cohort	MBC	Systemic therapy	Pre-treatment	35	15.0	64.0	IV	48.7(PNI)	OS
Yang, Z 2014 (37)	2003-2013	China	Retrospective cohort	TNBC	Surgery	Pre-treatment	382	74.0	50.0	I-III	48.7(PNI)	OS/DFS
Yildirim, S 2024a (42)	2010-2022	Turkey	Retrospective cohort	BC	NACT and surgery	Pre-treatment	624	42.0	50.0	I-III	52.7(PNI)	OS/DFS
Yildirim, S 2024b (42)	2010-2022	Turkey	Retrospective cohort	BC	NACT and surgery	Pre-treatment	624	42.0	50.0	I-III	54.0(PNI)	pCR
Zhang, X 2023a (45)	2012-2019	Multicenter	Prospective cohort	BC	NA	NA	1151	NA	52.5	I-IV	45.8(PNI)	OS
Zhang, X 2023b (45)	2012-2019	Multicenter	Prospective cohort	BC	NA	NA	1151	NA	52.5	I-IV	2.0(CONUT)	OS
Zhu, M 2022 (38)	2010-2016	China	Retrospective cohort	BC	Surgery	Pre-treatment	381	40.6	50.0	III	1.0(CONUT)	OS/DFS

### 3.2 Study quality

Across the 37 comparison groups, NOS scores ranged from 6 to 9 (**Supplementary Table 2**).

### 3.3 Meta-analysis results

#### 3.3.1 PNI and OS

**Figure 2:**
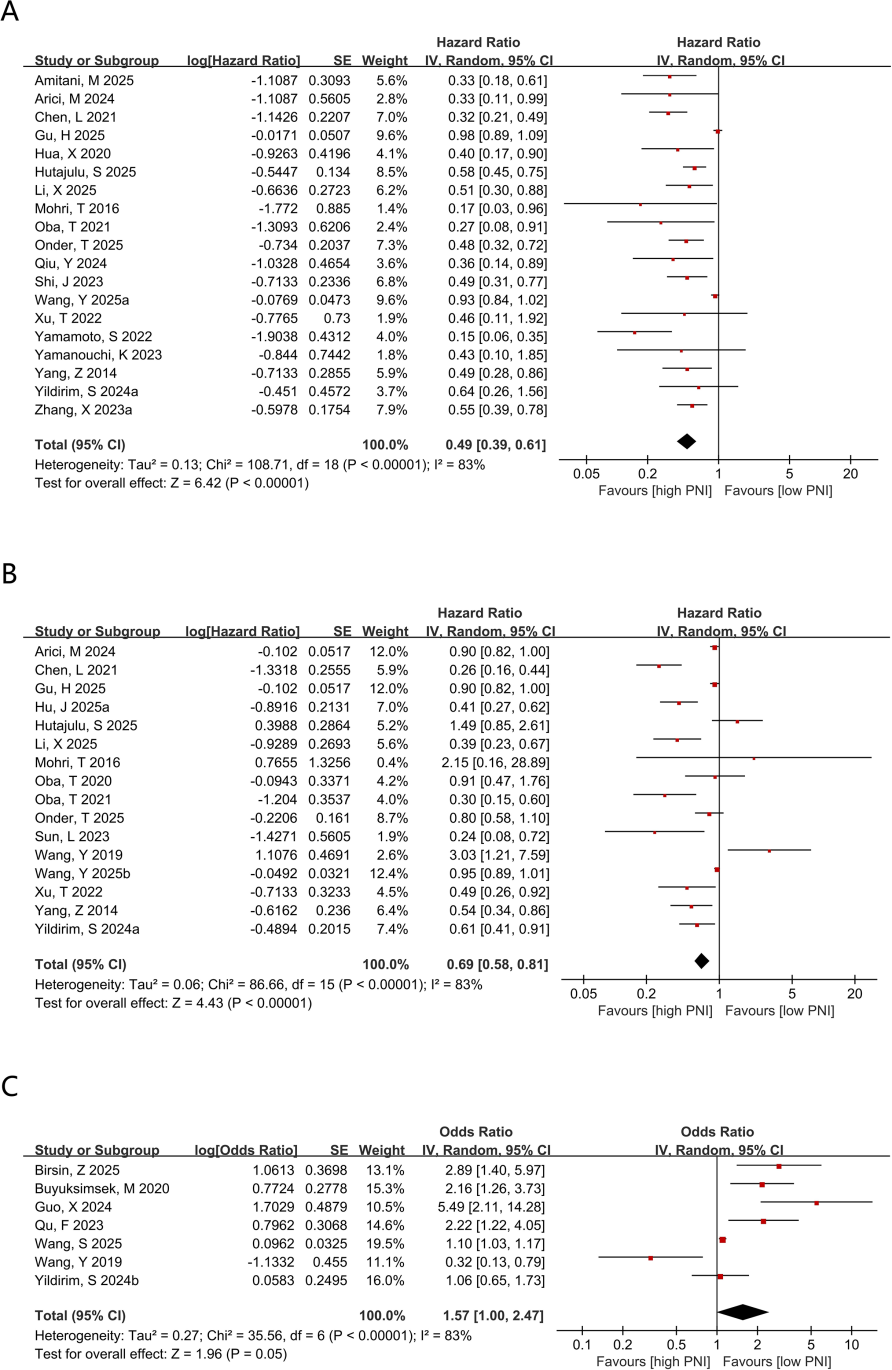
**(A)** Forest plots for the association between PNI and OS; **(B)** Forest plots for the association between PNI and DFS; **(C)** Forest plots for the association between PNI and pCR.

The association between PNI and OS was evaluated in 19 comparison groups, comprising 6,426 participants. The pooled estimate indicated that elevated PNI was associated with superior OS (HR = 0.49, 95% CI 0.39–0.61; P < 0.00001; **Figure 2A**). Substantial heterogeneity was observed (I² = 83%; τ² = 0.1426); therefore, a random-effects model was applied. The 95% prediction interval was 0.21–1.13, indicating that although the average pooled effect favored higher PNI, the magnitude of the effect may vary across future similar settings. To investigate the potential sources of heterogeneity, subgroup analyses were conducted. The association remained generally consistent across subgroups, and heterogeneity was attenuated in several strata (**Table 2**).

**Table 2:** Pooled HRs/ORs for OS, DFS and pCR in subgroup analyses of PNI.

Subgroup	OS				DFS				pCR			
	Study group	HR [95%CI]	*P* value	*I* ^2^	Study group	HR [95%CI]	*P* value	*I* ^2^	Study group	OR [95%CI]	*P* value	*I* ^2^
Total	19	0.49 [0.39, 0.61]	<0.00001	83%	16	0.69 [0.58, 0.81]	<0.00001	83%	7	1.57 [1.00, 2.47]	0.05	83%
Sample size												
<400	13	0.52 [0.40, 0.66]	<0.00001	83%	11	0.82 [0.70, 0.97]	0.02	76%	4	1.83 [0.67, 5.05]	0.24	86%
≥400	6	0.47 [0.39, 0.57]	<0.00001	0%	5	0.49 [0.34, 0.73]	0.0004	75%	3	1.26 [0.89, 1.80]	0.20	61%
Follow-up												
<40 months	8	0.42 [0.31, 0.55]	<0.00001	43%	6	0.81 [0.55, 1.21]	0.30	83%	NA	NA	NA	NA
≥40 months	9	0.61 [0.47, 0.79]	0.0002	81%	9	0.64 [0.51, 0.80]	<0.0001	85%	NA	NA	NA	NA
Mean/median Age												
<52	9	0.41 [0.30, 0.54]	<0.00001	42%	7	0.66 [0.46, 0.95]	0.03	83%	5	1.86 [1.12, 3.09]	0.02	82%
≥52	10	0.60 [0.47, 0.75]	<0.0001	82%	8	0.66 [0.52, 0.82]	0.0002	83%	1	2.16 [1.26, 3.73]	0.005	NA
Region												
Asia	13	0.51 [0.39, 0.66]	<0.00001	85%	13	0.64 [0.51, 0.80]	0.0001	85%	3	1.22 [0.39, 3.87]	0.73	89%
Non-Asia	4	0.44 [0.33, 0.59]	<0.00001	0%	3	0.82 [0.67, 1.00]	0.05	47%	2	1.50 [0.74, 3.02]	0.26	73%
Multicenter	2	0.53 [0.40, 0.69]	<0.00001	0%	NA	NA	NA	NA	2	2.47 [1.56, 3.92]	0.0001	0%
PNI cut-off												
<52	13	0.50 [0.39, 0.64]	<0.00001	87%	9	0.56 [0.44, 0.71]	<0.00001	88%	2	3.16 [1.29, 7.75]	0.01	64%
≥52	6	0.54 [0.43, 0.68]	<0.00001	0%	6	0.95 [0.65, 1.40]	0.81	71%	5	1.24 [0.78, 1.98]	0.37	79%
Tumor stage												
Metastatic	6	0.37 [0.26, 0.53]	<0.00001	35%	3	0.48 [0.26, 0.90]	0.02	79%	NA	NA	NA	NA
Non-metastatic	6	0.48 [0.27, 0.84]	0.01	85%	9	0.70 [0.55, 0.88]	0.002	85%	5	1.63 [0.76, 3.50]	0.21	84%
Mixed	6	0.53 [0.45, 0.64]	<0.00001	0%	2	0.86 [0.29, 2.57]	0.79	85%	2	1.46 [0.75, 2.87]	0.27	81%
Treatment method												
Surgery	7	0.77 [0.62, 0.96]	0.02	69%	7	0.70 [0.56, 0.87]	0.001	80%	NA	NA	NA	NA
Surgery and NACT	3	0.36 [0.25, 0.52]	<0.00001	0%	5	0.77 [0.45, 1.30]	0.32	88%	NA	NA	NA	NA
Other	7	0.42 [0.31, 0.57]	<0.00001	49%	4	0.63 [0.34, 1.16]	0.14	83%	NA	NA	NA	NA
Adjustment												
Univariate analysis	5	0.32 [0.24, 0.44]	<0.00001	0%	5	0.79 [0.43, 1.42]	0.43	85%	1	1.10 [1.03, 1.17]	0.003	NA
Multivariate analysis	14	0.56 [0.45, 0.70]	<0.00001	82%	11	0.65 [0.53, 0.80]	<0.0001	83%	6	1.72 [0.92, 3.20]	0.09	81%

Univariable meta-regression analyses were further conducted, and residual heterogeneity (τ²) together with pseudo-R² was recorded. Treatment modality and adjustment status showed the most consistent heterogeneity-explaining ability. In univariable meta-regression, treatment modality was significantly associated with the pooled effect (NACT and surgery vs surgery: coefficient = -0.949, 95% CI -1.322 to -0.576, P < 0.001; other vs surgery: coefficient = -0.664, 95% CI -0.860 to -0.468, P < 0.001), and adjustment status was also significant (coefficient = 0.550, 95% CI 0.055 to 1.045, P = 0.030) (**Table 3**). Tumor stage reduced the residual heterogeneity from τ² = 0.1426 to τ² = 0.095 (pseudo-R² = 33.66%), although the category-specific coefficients were not statistically significant. In the primary multivariable meta-regression model including treatment modality and adjustment status, residual heterogeneity was reduced to τ² = 0 (pseudo-R² = 100%) (**Table 4**). In addition, an exploratory multivariable model that further included tumor stage was provided in **Supplementary Table 5** and should be interpreted cautiously because of the limited number of studies and the possibility of model overfitting. Finally, to reduce potential confounding, only studies reporting HRs derived from multivariable Cox regression analyses were included in the pooled analysis. The pooled HR remained highly significant (HR = 0.56, 95% CI 0.45–0.70; P < 0.00001) (**Supplementary Figure 1A**).

**Table 3:** Univariate meta-regression for the association of confounding factors and the hazard ratio for OS.

Variables	Coefficient	P value	95% CI	R²	τ²
Sample size					
Per 1-patient increase	0.00010	0.808	[-0.00069,0.00088]	-9.40%	0.156
Follow-up					
Per 1-month increase	0.008	0.238	[-0.005,0.022]	-15.64%	0.165
Mean/Median Age					
Per 1-year increase	0.014	0.635	[-0.043,0.071]	0.63%	0.142
PNI cut-off					
Per 1-unit increase	0.008	0.802	[-0.052,0.067]	-7.50%	0.153
Region					
Non-Asia vs Asia	-0.128	0.676	[-0.730,0.473]	-10.66%	0.158
Multicenter vs Asia	0.062	0.859	[-0.622,0.746]		
Tumor stage					
Non-metastatic vs Metastatic	0.372	0.166	[-0.155,0.900]	33.66%	0.095
Mixed vs Metastatic	0.312	0.238	[-0.207,0.831]		
Treatment method					
NACT and Surgery vs Surgery	-0.949	<0.001	[-1.322,-0.576]	100%	0
Other vs Surgery	-0.664	<0.001	[-0.860,-0.468]		
Adjustment					
Multivariate vs Univariate	0.550	0.030	[0.055,1.045]	31.70%	0.097

**Table 4:** Multivariable meta-regression for the association of confounding factors and the hazard ratio for OS.

Variables	Coefficient	P value	95% CI	R²	τ²
Treatment method					
NACT and Surgery vs Surgery	-0.434	0.101	[-0.954,0.085]	100%	0
Other vs Surgery	-0.596	<0.001	[-0.797,-0.394]		
Adjustment					
Multivariate vs Univariate	0.623	0.005	[0.186,1.059]		

#### 3.3.2 PNI and DFS

Data on PNI and DFS were available from 16 comparison groups. Random-effects meta-analysis showed that a higher PNI was significantly associated with longer DFS (HR = 0.69, 95% CI 0.58–0.81; P < 0.00001; **Figure 2B**). Substantial heterogeneity was present (I² = 83%; τ² = 0.261), and the 95% prediction interval was 0.23–2.10, indicating considerable between-study variability in the expected effect across future settings. Subsequent subgroup analyses showed that no significant prognostic value of PNI for DFS was observed in several subgroups, whereas significant associations were retained in the remaining subgroups (**Table 2**).

Univariable meta-regression analyses were conducted for sample size, follow-up duration, mean/median age, cutoff value, region, tumor stage, treatment modality, and adjustment status (**Table 5**). None of these covariates showed a statistically significant association with the pooled effect. Most moderators yielded negative or near-zero pseudo-R² values, indicating little or no explanatory value for the observed heterogeneity. Mean/median age reduced the residual heterogeneity from τ² = 0.261 to τ² = 0.196 (pseudo-R² = 24.90%), but the coefficient remained non-significant (P = 0.367). Given the limited number of studies and the absence of moderators showing both statistical significance and consistent heterogeneity-explaining ability, formal multivariable meta-regression was not pursued in order to avoid model overfitting. Finally, to minimize potential confounding, only studies reporting HRs derived from multivariable Cox regression analyses were included in the pooled analysis. The pooled HR remained highly significant (HR = 0.65, 95% CI 0.53–0.80; P < 0.0001) (**Supplementary Figure 1B**).

**Table 5:** Univariate meta-regression for the association of confounding factors and the hazard ratio for DFS.

Variables	Coefficient	P value	95% CI	R²	τ²
Sample size					
Per 1-patient increase	-0.00084	0.333	[-0.00253,0.00086]	2.53%	0.254
Follow-up					
Per 1-month increase	-0.004	0.558	[-0.019,0.010]	-2.03%	0.266
Mean/Median Age					
Per 1-year increase	0.037	0.367	[-0.043,0.117]	24.90%	0.196
PNI cut-off					
Per 1-unit increase	0.073	0.189	[-0.036,0.182]	-11.34%	0.291
Region					
Non-Asia vs Asia	0.197	0.584	[-0.510,0.905]	-6.86%	0.279
Tumor stage					
Non-metastatic vs Metastatic	0.408	0.322	[-0.400,1.216]	-17.20%	0.306
Mixed vs Metastatic	0.600	0.293	[-0.517,1.717]		
Treatment method					
Surgery+NACT vs Surgery	0.246	0.515	[-0.494,0.986]	-24%	0.323
Other vs Surgery	0.043	0.916	[-0.746,0.831]		
Adjustment					
Multivariate vs Univariate	-0.239	0.481	[-0.903,0.425]	-8.28%	0.283

#### 3.3.3 PNI and pCR

The association between PNI and pCR was evaluated in seven comparison groups comprising 2,948 patients. Substantial heterogeneity was present (I² = 83%; τ² = 0), and the 95% prediction interval was 0.87–2.84, indicating uncertainty in the effect expected in a future similar study. Under a random-effects model, the primary pooled analysis did not demonstrate a statistically significant association between PNI and pCR (OR = 1.57, 95% CI 1.00-2.47; P = 0.05; **Figure 2C**). Because the median age was not reported in the study by Wang et al. (18), age-stratified subgroup analyses were conducted using the remaining six groups. The results indicated that a higher PNl was significantly associated with a higher pCR rate both in cohorts with a median age<52 years (OR = 1.86, 95% CI 1.12-3.09; P = 0.02) and those with a median age > 52 years (OR = 2.16, 95% CI 1.26-3.73; P = 0.005). Moreover, a higher PNI was significantly associated with an increased pCR rate in breast cancer patients in the multicenter subgroup (OR = 2.47, 95% CI 1.56-3.92; P = 0.0001), the subgroup with a cutoff value < 52 (OR = 3.16, 95% CI 1.29-7.75; P = 0.01), and the univariable-analysis subgroup (OR = 1.10, 95% CI 1.03-1.17; P = 0.003). In the remaining subgroups, however, no significant association was observed between pretreatment PNI and pCR. Because all included patients were treated with NACT followed by surgery, and because only a limited number of studies reported follow-up duration, subgroup analyses according to treatment modality and follow-up duration were not conducted. In addition, heterogeneity analysis identified region as a source of heterogeneity. In addition, subgroup analyses suggested that region might have contributed to the observed heterogeneity. However, meta-regression was not performed because only seven comparison groups were available and several moderator categories contained very sparse data, making meta-regression estimates statistically unreliable and highly susceptible to overfitting. Therefore, heterogeneity for pCR was explored using subgroup analyses, sensitivity analyses, and prediction intervals rather than formal meta-regression. The pooled OR from multivariable-adjusted studies was not statistically significant (OR = 1.72, 95% CI 0.92-3.20; P = 0.09) (**Supplementary Figure 1C**), suggesting that the evidence for an association between PNI and pCR remained inconclusive.

#### 3.3.4 CONUT and OS

**Figure 3:**
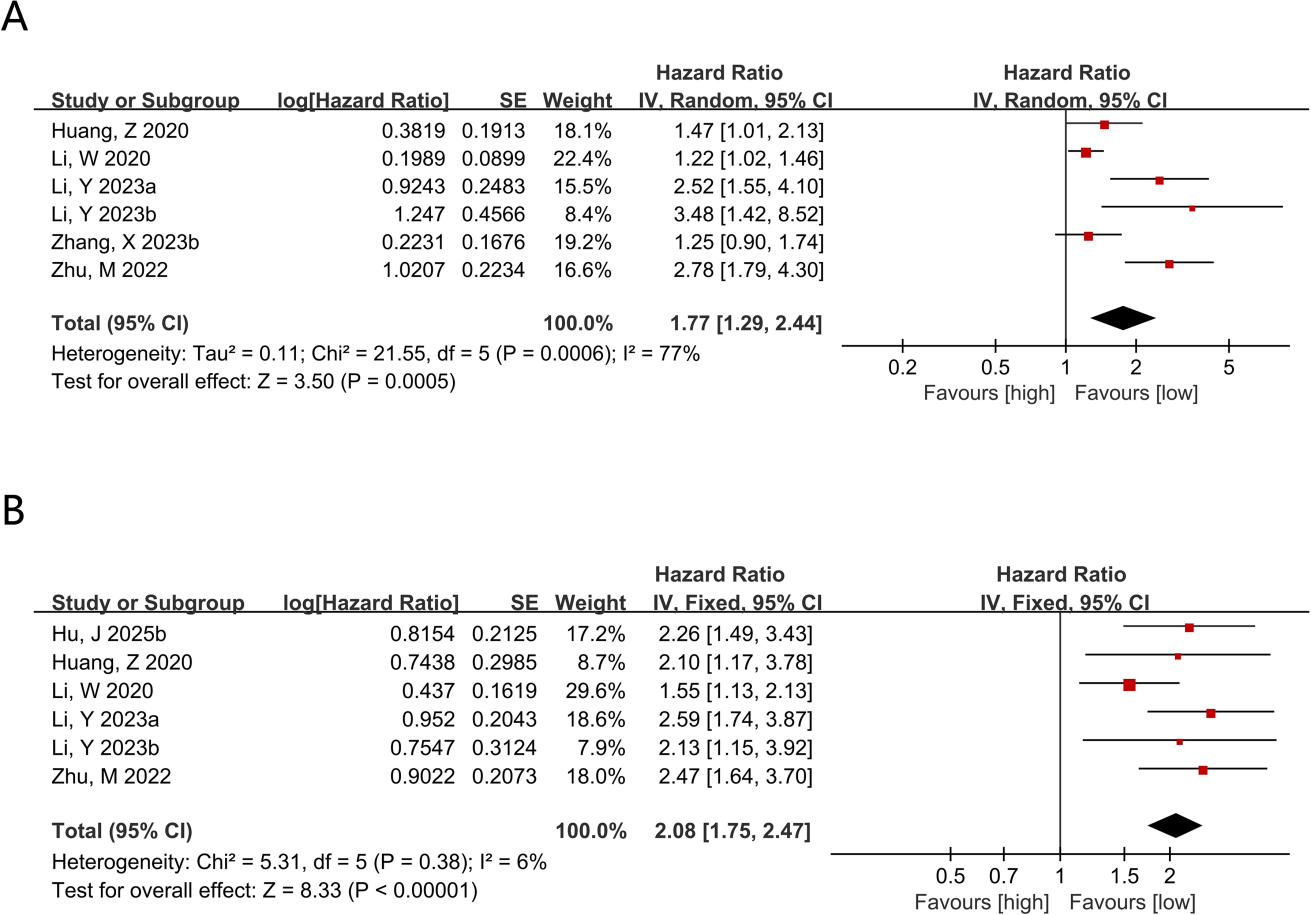
**(A)** Forest plots for the association between CONUT and OS; **(B)** Forest plots for the association between CONUT and DFS.

Data on CONUT and OS were available from 6 comparison groups, and multivariable-adjusted hazard ratios were reported in all included studies. Under a random-effects model, higher CONUT was linked with inferior OS compared with lower CONUT (HR = 1.77, 95% CI 1.29–2.44; P = 0.0005; **Figure 3A**). The 95% prediction interval was 0.60–5.24, indicating that the expected effect in a future similar study may vary substantially despite the significant average pooled estimate. Subgroup analyses stratified by sample size, age, cutoff value, and tumor stage were conducted to explore effect modification and heterogeneity. No significant association was identified in studies using a cutoff <3 (P = 0.13), whereas significant associations were observed in the remaining subgroups. Heterogeneity was mainly attributable to differences in sample size and clinical stage (**Table 6**). Given the limited number of studies assessing the association between CONUT and OS, meta-regression analysis was not conducted.

**Table 6:** Pooled HRs/ORs for OS, DFS and pCR in subgroup analyses of CONUT.

Subgroup	OS				DFS			
	Study group	HR [95%CI]	*P* value	*I* ^2^	Study group	HR [95%CI]	*P* value	*I* ^2^
Total	6	1.77 [1.29, 2.44]	0.0005	77%	6	2.08 [1.75, 2.47]	<0.00001	6%
Sample size								
<400	2	2.90 [1.96, 4.30]	<0.00001	0%	2	2.36 [1.68, 3.31]	<0.00001	0%
≥400	4	1.45 [1.11, 1.88]	0.006	62%	4	2.03 [1.58, 2.62]	<0.00001	33%
Mean/median Age								
<52	2	2.00 [1.07, 3.73]	0.03	79%	2	2.34 [1.68, 3.27]	<0.00001	0%
≥52	4	1.66 [1.13, 2.45]	0.01	75%	4	2.00 [1.63, 2.44]	<0.00001	33%
CONUT cut-off								
<3	2	1.84 [0.84, 4.01]	0.13	88%	2	2.36 [1.77, 3.16]	<0.00001	0%
≥3	4	1.76 [1.17, 2.64]	0.006	75%	4	1.94 [1.57, 2.41]	<0.00001	27%
Tumor stage								
Non-metastatic	3	2.74 [2.02, 3.72]	<0.00001	0%	4	2.40 [1.92, 2.99]	<0.00001	0%
Mixed	2	1.34 [1.05, 1.71]	0.02	0%	1	2.10 [1.17, 3.78]	0.01	NA

#### 3.3.5 CONUT and DFS

Data on CONUT and DFS were available from 6 comparison groups, and multivariable-adjusted hazard ratios were reported in all included studies. Using a fixed-effect model, higher CONUT was linked with shorter DFS (HR = 2.08, 95% CI 1.75–2.47; P < 0.00001; **Figure 3B**). Subgroup analyses indicated that this association remained statistically significant in each subgroup. Heterogeneity was mainly attributable to differences in sample size and follow-up duration (**Table 6**). Likewise, meta-regression analysis was not conducted for this part. No eligible comparison group reported outcomes for CONUT and pCR; therefore, pooled analyses and subgroup analyses for CONUT–pCR were not performed.

### 3.4 Sensitivity analysis

**Figure 4:**
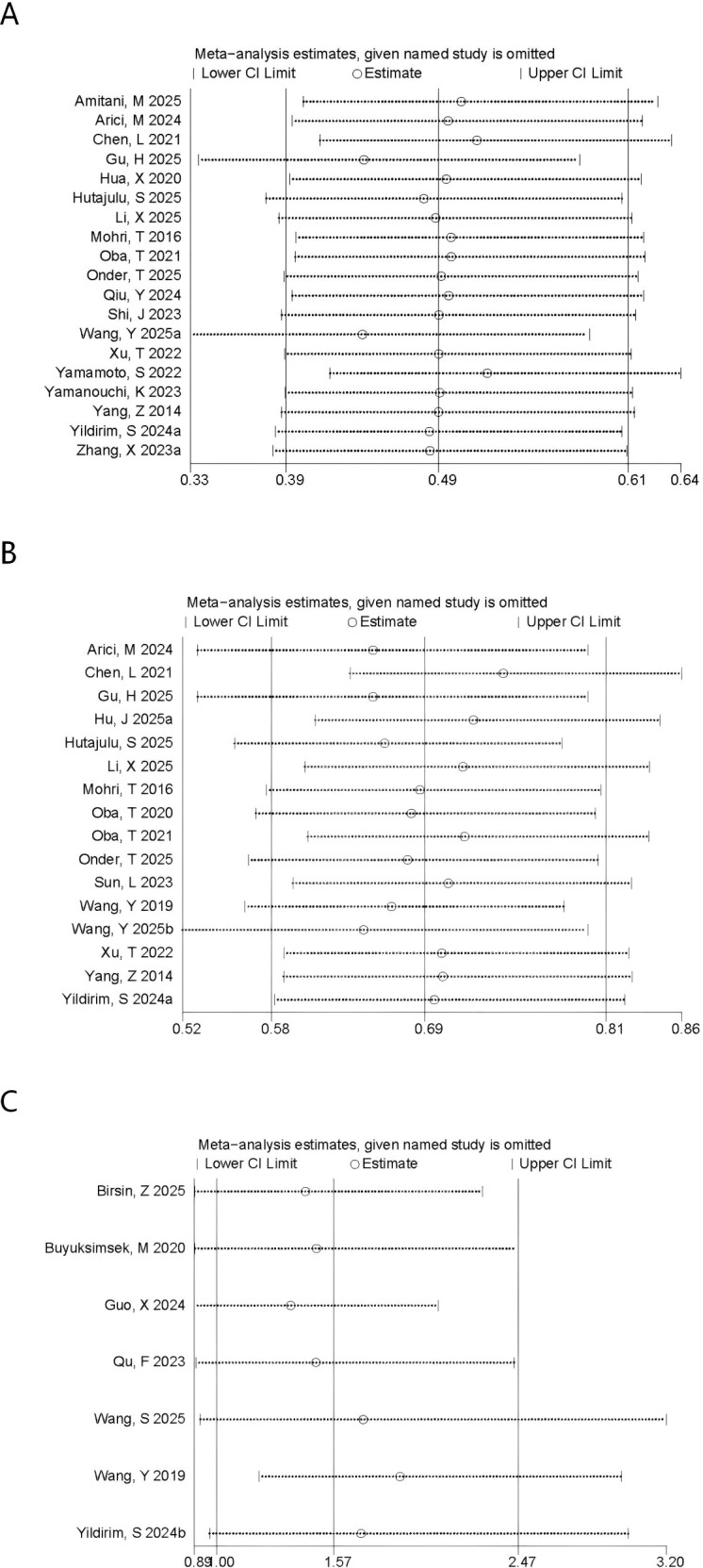
Sensitivity analysis of **(A)** OS **(B)** DFS and **(C)** pCR for PNI.

**Figure 5:**
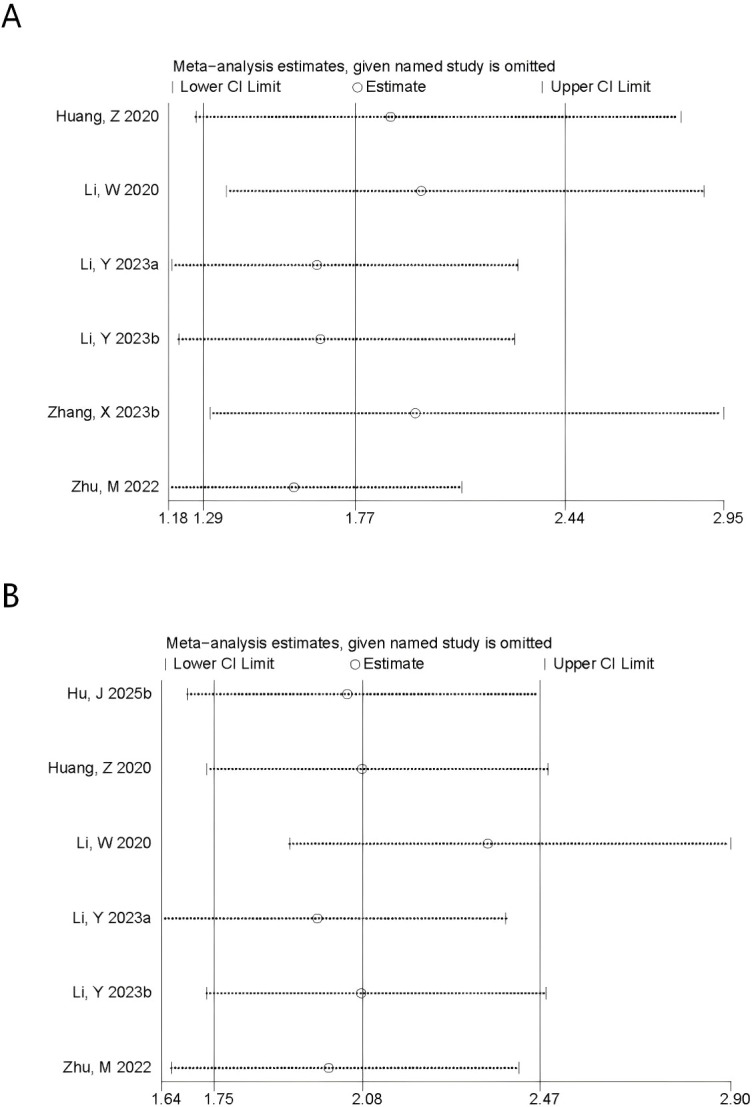
Sensitivity analysis of **(A)** OS and **(B)** DFS for CONUT.

Sensitivity analyses were performed to appraise the robustness of the prognostic associations for PNI and CONUT in breast cancer. First, studies were sequentially excluded one by one, and the results for PNI showed that no single included study materially affected the overall results for OS (**Figure 4A**) or DFS (**Figure 4B**). However, when evaluating the association between PNl and pCR (**Figure 4C**), removal of the study by Wang et al. (18) yielded a materially different pooled estimate (OR = 1.90, 95% CI 1.21-2.98), with a confidence interval that no longer overlapped the original estimate. These findings suggested that, although the primary analysis did not demonstrate a statistically significant association between PNI and pCR (P = 0.05), the result was sensitive to individual studies and was therefore less stable. Accordingly, the evidence for an association between PNI and pCR remained inconclusive and appeared to be sensitive to the influence of individual studies. For CONUT, the pooled estimates for OS (**Figure 5A**) and DFS (**Figure 5B**) remained stable, with no single comparison group exerting a disproportionate influence. Overall, robustness was supported for OS and DFS, whereas the association between PNI and pCR appeared more sensitive to individual-study effects. Furthermore, to reduce the potential influence of clinical confounding factors, sensitivity analyses were conducted by including only HRs derived from multivariable-adjusted models. After studies reporting univariable estimates were excluded, the prognostic significance of PNI and CONUT for OS and DFS remained significant, further confirming the robustness of the pooled findings.

### 3.5 Publication bias

Funnel plots and Egger’s test were used to evaluate publication bias for the associations of PNI with OS and DFS. Egger’s test indicated potential publication bias for both OS (P < 0.001) and DFS (P = 0.026). To account for this possibility, trim-and-fill analyses were performed; however, no definite missing studies were imputed, and no “filled” studies were added. After trim-and-fill adjustment, the pooled estimates remained essentially unchanged for OS (HR = 0.49, 95% CI 0.40-0.61; P < 0.00001; **Supplementary Figure 2A**) and DFS (HR = 0.69, 95% CI 0.58-0.81; P < 0.00001; **Supplementary Figure 2B**). Although the trim-and-fill method did not identify any potentially missing studies, the influence of small-study effects could not be completely ruled out, given the substantial heterogeneity and the predominance of retrospective cohort studies. To further assess the robustness of the findings, additional validation was performed using subgroup analyses and meta-regression. In the large-sample subgroup (sample size ≥ 400), the prognostic significance of PNI for OS remained evident (HR = 0.47, 95% CI 0.39-0.57), and heterogeneity was eliminated (I² = 0%). Moreover, sensitivity analyses restricted to multivariable-adjusted estimates yielded consistent results, further supporting the robustness of the pooled findings. Collectively, these findings suggested that, although some risk of publication bias remained, the association between a higher PNI and more favorable survival outcomes was consistently observed in high-quality studies, large-sample studies, and studies with multivariable adjustment, indicating that the likelihood of substantial distortion by small-study effects remained limited.

**Figure 6:**
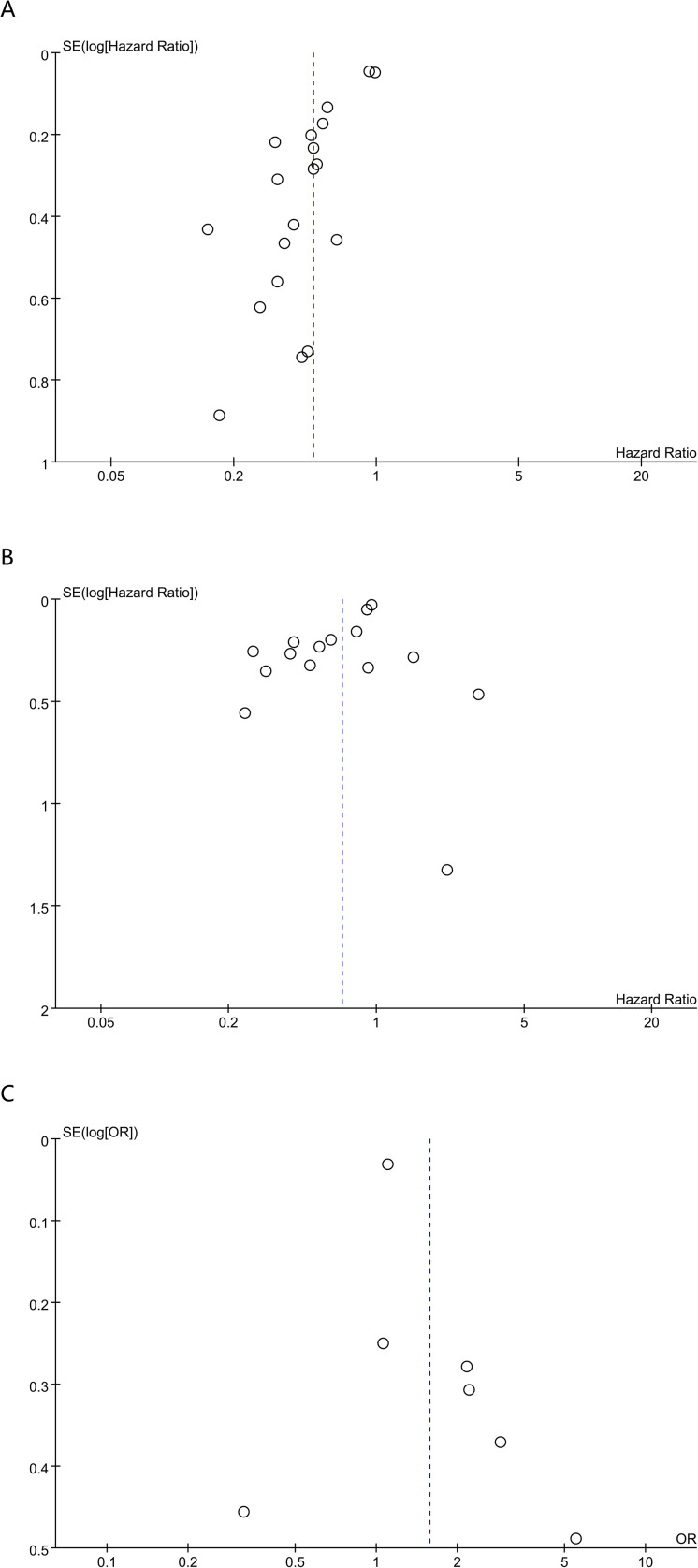
Funnel plot for the evaluation of publication bias for **(A)** OS **(B)** DFS and **(C)** pCR in PNI.

**Figure 7:**
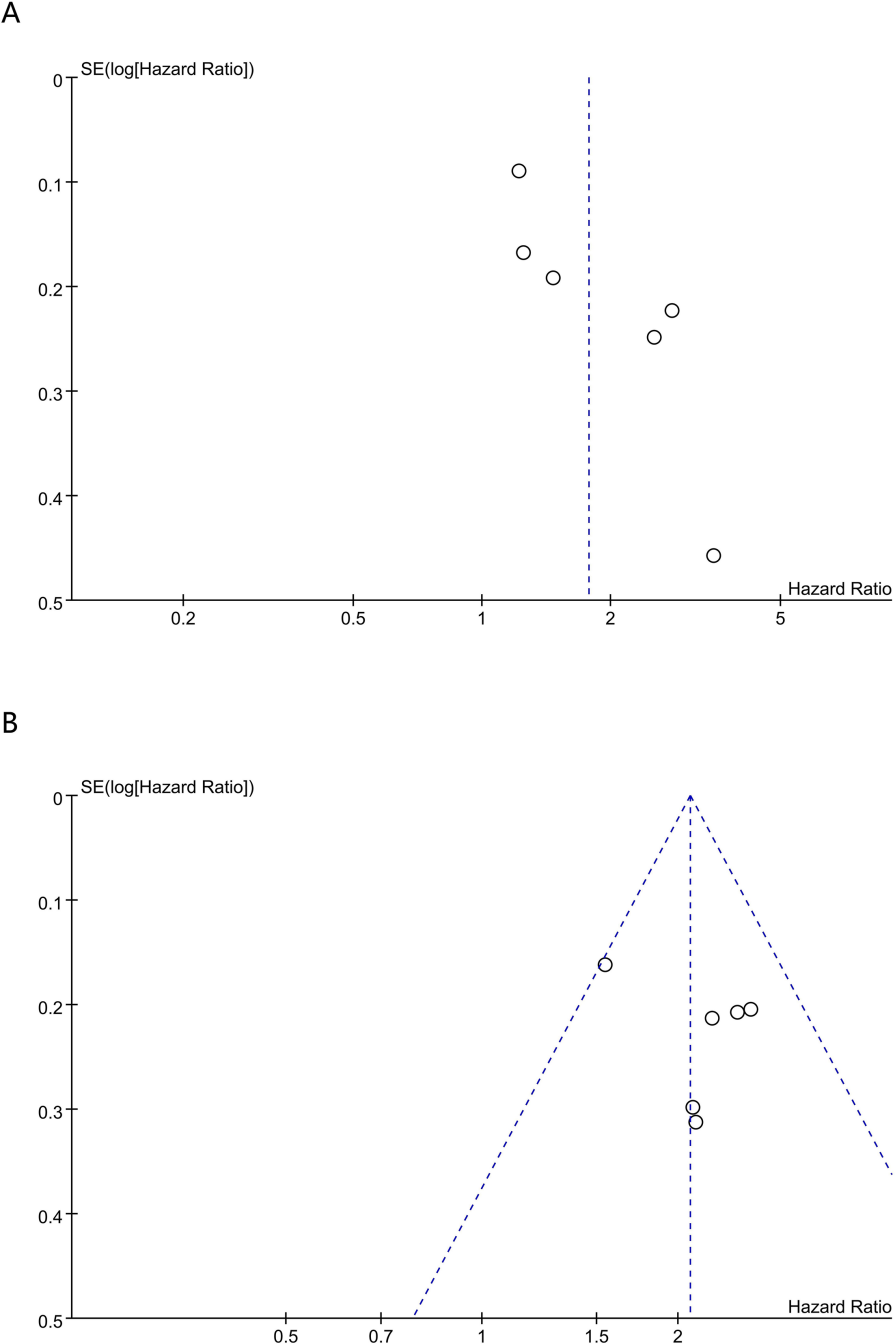
Funnel plot for the evaluation of publication bias for **(A)** OS and **(B)** DFS in CONUT.

Because Egger’s test was generally applied when more than 10 studies were selected, publication bias for the associations between PNI and pCR and between CONUT and OS and DFS was evaluated via funnel plots and Begg’s test. No evidence of publication bias was noted for PNI and pCR (Begg’s P = 0.236), CONUT and OS (Begg’s P = 0.707), or CONUT and DFS (Begg’s P = 0.383). Qualitative assessment of funnel plots corroborated these tests, suggesting publication bias for PNI with OS (**Figure 6A**) and DFS (**Figure 6B**), but no publication bias for pCR (**Figure 6C**), and no publication bias for CONUT with OS (**Figure 7A**) and DFS (**Figure 7B**).

## 4 Discussion

In this meta-analysis, both the PNI and CONUT score were shown to be prognostic biomarkers in breast cancer. Higher PNI was significantly linked with improved OS and DFS, whereas higher CONUT was correlated with worse OS and DFS. Although the primary pooled analysis did not demonstrate a statistically significant association between PNI and pCR, sensitivity analyses indicated that this finding was unstable. Therefore, the current evidence for an association between PNI and pCR should be considered inconclusive and sensitive to individual studies. Overall, sensitivity analyses further supported the robustness of the prognostic associations of PNI and CONUT for OS and DFS, while Egger’s and Begg’s tests suggested a risk of publication bias in the analyses of PNI with OS and DFS. To further reduce the potential influence of clinical confounding factors, sensitivity analyses were conducted by restricting the analysis to multivariable-adjusted estimates. The results supported the overall prognostic value of both PNI and CONUT. However, the pooled findings should still be interpreted with caution because residual confounding could not be fully excluded and the cutoff definitions for PNI and CONUT were not uniform across studies.

Compared with the previous meta-analyses by Prasetiyo et al. (15, 46), the present study provides several methodological advantages. While earlier studies found that a higher PNI was associated with better OS but not DFS, our analysis showed that PNI was also significantly associated with DFS. This difference may be explained by the inclusion of more up-to-date evidence through November 2025, comprising 15 studies with a larger pooled sample size and a more diverse population, including multicenter and non-Asian cohorts. In addition, this study used a more comprehensive analytical framework. Subgroup analyses were performed according to region, sample size, cutoff values, follow-up duration, mean age, and tumor stage, allowing a more detailed evaluation of the prognostic relevance of PNI and CONUT. More importantly, hazard ratios from multivariable Cox regression models were preferentially synthesized when available, which improved adjustment for potential confounders. Univariable and multivariable meta-regression analyses were further performed for PNI and OS. When residual heterogeneity (τ²) and explained variance (pseudo-R²) were considered together, treatment modality and adjustment status showed the most consistent heterogeneity-explaining ability. Tumor stage also reduced τ² in univariable analysis, although its category-specific coefficients did not reach statistical significance and were therefore interpreted cautiously.

The sources of heterogeneity were further assessed by subgroup analyses and meta-regression. Stratification by sample size, geographic region, tumor stage, treatment modality, and adjustment status substantially reduced heterogeneity in several subgroups, and in the analysis of PNI and OS, heterogeneity was eliminated in some strata. In univariable meta-regression for OS, treatment modality and adjustment status were the most consistent moderators, whereas tumor stage showed a moderate reduction in residual heterogeneity but without statistically significant category-specific coefficients. In contrast, for DFS, most covariates yielded negative or negligible pseudo-R² values, suggesting limited capacity to explain the observed heterogeneity. Moreover, the 95% prediction intervals for PNI and OS, PNI and DFS, PNI and pCR, and CONUT and OS all crossed the null value, indicating that although the pooled effects were statistically significant on average, the magnitude of the effect may vary across future study settings.

Because PNI and CONUT are derived from serum albumin, total cholesterol, and peripheral lymphocyte counts, their prognostic relevance may be explained by the physiological roles of these components. Albumin is a major visceral protein that is commonly used as a surrogate marker of protein reserves and is involved in maintaining colloid osmotic pressure, transporting endogenous and exogenous small molecules, and providing antioxidant activity (47). During malignant progression, sustained hyperproliferation and hypermetabolism occur, and amino acid and energy demands are increased; accelerated albumin uptake is also observed within the tumor microenvironment, potentially to compensate for amino acid scarcity (48). In parallel, tumor-associated inflammation and tissue injury are accompanied by cytokine-mediated suppression of hepatic albumin synthesis and enhanced proteolysis, leading to persistently reduced serum albumin levels (49). Experimental tumor systems have reported that as tumors expand, albumin gene transcription in the liver declines and low serum albumin levels emerge (50). Over time, the gap widens between production and utilization, creating a profile compatible with nutritional depletion and ongoing inflammatory activity. Alongside this, redox buffering capacity is reduced and fewer circulating proteins are available to bind antineoplastic compounds, with downstream effects on pharmacokinetics, tissue healing, and the ability to sustain therapy—all of which are factors that can ultimately worsen clinical outcomes (51).

High lymphocytic infiltration in breast cancer has been reported to be closely linked with a favorable prognosis (52). The peripheral lymphocyte count is widely used as a marker of host cellular immune competence (53). Cytotoxic CD8^+^ T cells mediate direct tumor cell killing through secretion of perforin, granzyme B, and other effector molecules (54), whereas CD4^+^ helper T cells enhance antitumor immunity by producing cytokines such as interferon-γ (IFN-γ) and by promoting the activation and maintenance of effector T cells (55). Reduced peripheral lymphocyte counts are frequently accompanied by fewer tumor-infiltrating lymphocytes (TILs), which compromises immune surveillance of tumor growth and metastatic spread, diminishes responsiveness to cytotoxic therapies (e.g., chemotherapy and radiotherapy), and is associated with shortened survival (56). Consistently, *in vivo* breast tumor models have indicated that increasing tumor burden is accompanied by progressive lymphocyte depletion and reduced survival (57). Total cholesterol is also a key structural component of cellular membranes and lipid rafts and is required for T cell receptor clustering, immune synapse formation, and cytokine signal transduction; adequate cholesterol availability therefore supports effective antitumor immune function (58). Conversely, sustained malnutrition and tumor-associated catabolism can reduce circulating lipid levels, and hypocholesterolemia often co-occurs with depleted nutritional reserves and chronic systemic inflammation. Collectively, low PNI or high CONUT is associated with a biological milieu characterized by impaired immune competence and nutritional depletion, which may facilitate more rapid tumor progression and dissemination and thereby increase recurrence risk and worsen prognosis.

First, substantial between-study heterogeneity was observed, particularly in the analyses of PNI with OS, DFS, and pCR and of CONUT with OS, which may reduce the robustness and generalizability of the pooled estimates. Although subgroup analyses, meta-regression, sensitivity analyses, and prediction intervals were used to explore heterogeneity, residual confounding and unexplained between-study variability cannot be fully eliminated. Importantly, the 95% prediction intervals for PNI with OS, DFS, and pCR and for CONUT with OS all crossed the null value, suggesting that the magnitude of the observed associations may vary across future clinical settings. In addition, because the number of available studies was limited for several outcomes, especially pCR and CONUT-related analyses, the power of subgroup analyses and meta-regression to identify stable moderators remained restricted. Breast cancer is molecularly heterogeneous. However, because most original studies did not report subtype-specific data, subgroup analyses could not be conducted, limiting the generalizability of the findings to specific biological subtypes. Second, most included studies were retrospective, which may have introduced selection bias, immortal time bias, and treatment-related confounding, thereby limiting causal inference. In addition, PNI and CONUT cutoff values are not standardized. In several studies, thresholds were adopted from prior literature rather than being derived using receiver operating characteristic (ROC) analyses. Even when ROC methods were used, cutoff values may vary by population, baseline laboratory distributions, specimen handling, and the timing of biomarker assessment, which can introduce selection bias and limit comparability across studies. Accordingly, the pooled effect estimates should be interpreted cautiously, because part of the observed associations may depend on how high and low groups were defined in the original studies. To improve reliability and facilitate cross-study comparison, standardized and clinically validated cutoff values for PNI and CONUT are needed in future investigations.

## 5 Conclusion

This meta-analysis indicates that both the PNI and CONUT score significantly predict outcomes in breast cancer. Patients with higher PNI tend to experience better OS and DFS, while those with higher CONUT show the opposite pattern. This pattern indicates that both indices provide prognostic information beyond routine clinical factors and points to host nutritional and immune status as a modifiable avenue for intervention. However, given the inherent limitations of the included studies and the lack of molecular subtype data, further prospective studies are needed to validate our findings across different racial and geographic populations.

## Data Availability

The original contributions presented in the study are included in the article/**Supplementary Material**. Further inquiries can be directed to the corresponding author.

## References

[1] Bray F Laversanne M Sung H Ferlay J Siegel RL Soerjomataram I . Global cancer statistics 2022: GLOBOCAN estimates of incidence and mortality worldwide for 36 cancers in 185 countries. CA Cancer J Clin. (2024) 74:229–63. doi: 10.3322/caac.21834. PMID: 38572751

[2] Dowsett M Cuzick J Wale C Forbes J Mallon EA Salter J . Prediction of risk of distant recurrence using the 21-gene recurrence score in node-negative and node-positive postmenopausal patients with breast cancer treated with anastrozole or tamoxifen: a TransATAC study. J Clin Oncol. (2010) 28:1829–34. doi: 10.1200/jco.2009.24.4798. PMID: 20212256

[3] Cullinane C Fleming C O’Leary DP Hassan F Kelly L O’Sullivan MJ . Association of circulating tumor DNA with disease-free survival in breast cancer: a systematic review and meta-analysis. JAMA Netw Open. (2020) 3:e2026921. doi: 10.1001/jamanetworkopen.2020.26921. PMID: 33211112 PMC7677763

[4] Zhao H Wu L Yan G Chen Y Zhou M Wu Y . Inflammation and tumor progression: signaling pathways and targeted intervention. Signal Transduct Target Ther. (2021) 6:263. doi: 10.1038/s41392-021-00658-5. PMID: 34248142 PMC8273155

[5] Mireştean CC Stan MC Iancu RI Iancu DPT Bădulescu F . The prognostic value of platelet-lymphocyte ratio, neutrophil-lymphocyte ratio, and monocyte-lymphocyte ratio in head and neck squamous cell carcinoma (HNSCC)-a retrospective single center study and a literature review. Diagnostics (Basel). (2023) 13:3396. doi: 10.3390/diagnostics13223396. PMID: 37998532 PMC10670617

[6] Tan S Zheng Q Zhang W Zhou M Xia C Feng W . Prognostic value of inflammatory markers NLR, PLR, and LMR in gastric cancer patients treated with immune checkpoint inhibitors: a meta-analysis and systematic review. Front Immunol. (2024) 15:1408700. doi: 10.3389/fimmu.2024.1408700. PMID: 39050856 PMC11266030

[7] Shi J Liu T Ge Y Liu C Zhang Q Xie H . Cholesterol-modified prognostic nutritional index (CPNI) as an effective tool for assessing the nutrition status and predicting survival in patients with breast cancer. BMC Med. (2023) 21:512. doi: 10.1186/s12916-023-03225-7. PMID: 38129842 PMC10740286

[8] Van Cutsem E Arends J . The causes and consequences of cancer-associated malnutrition. Eur J Oncol Nurs. (2005) 9:S51–63. doi: 10.1016/j.ejon.2005.09.007. PMID: 16437758

[9] Yan L Nakamura T Casadei-Gardini A Bruixola G Huang YL Hu ZD . Long-term and short-term prognostic value of the prognostic nutritional index in cancer: a narrative review. Ann Transl Med. (2021) 9:1630. doi: 10.21037/atm-21-4528. PMID: 34926674 PMC8640913

[10] Kim SI Kim SJ Kim SJ Cho DS . Prognostic nutritional index and prognosis in renal cell carcinoma: a systematic review and meta-analysis. Urol Oncol. (2021) 39:623–30. doi: 10.1016/j.urolonc.2021.05.028. PMID: 34253447

[11] Chen L Bai P Kong X Huang S Wang Z Wang X . Prognostic nutritional index (PNI) in patients with breast cancer treated with neoadjuvant chemotherapy as a useful prognostic indicator. Front Cell Dev Biol. (2021) 9:656741. doi: 10.3389/fcell.2021.656741. PMID: 33859986 PMC8042235

[12] Oba T Maeno K Ono M Ito T Kanai T Ito KI . Prognostic nutritional index is superior to neutrophil-to-lymphocyte ratio as a prognostic marker in metastatic breast cancer patients treated with eribulin. Anticancer Res. (2021) 41:445–52. doi: 10.21873/anticanres.14794. PMID: 33419842

[13] Kheirouri S Alizadeh M . Prognostic potential of the preoperative controlling nutritional status (CONUT) score in predicting survival of patients with cancer: a systematic review. Adv Nutr. (2021) 12:234–50. doi: 10.1093/advances/nmaa102. PMID: 32910812 PMC7850023

[14] Huang ZZ Song CG Huang JJ Xia W Bi XW Hua X . Prognostic significance of the controlling nutritional status (CONUT) score in surgically treated breast cancer patients. Gland Surg. (2020) 9:1370–9. doi: 10.21037/gs-20-294. PMID: 33224812 PMC7667092

[15] Prasetiyo PD Baskoro BA Hariyanto TI . The role of nutrition-based index in predicting survival of breast cancer patients: a systematic review and meta-analysis. Heliyon. (2024) 10:e23541. doi: 10.1016/j.heliyon.2023.e23541. PMID: 38169970 PMC10758813

[16] Page MJ McKenzie JE Bossuyt PM Boutron I Hoffmann TC Mulrow CD . The PRISMA 2020 statement: an updated guideline for reporting systematic reviews. BMJ. (2021) 372:n71. doi: 10.1136/bmj.n71. PMID: 33782057 PMC8005924

[17] Amitani M Oba T Kitazawa A Iji R Kiyosawa N Katsuyama S . Prognostic significance of baseline skeletal muscle index and its dynamics in patients with metastatic breast cancer undergoing eribulin treatment. Breast Cancer Res Treat. (2025) 214:419–29. doi: 10.1007/s10549-025-07827-y. PMID: 41091279 PMC12583309

[18] Wang Y Battseren B Yin W Lin Y Zhou L Yang F . Predictive and prognostic value of prognostic nutritional index for locally advanced breast cancer. Gland Surg. (2019) 8:618–26. doi: 10.21037/gs.2019.10.08. PMID: 32042668 PMC6989918

[19] Higgins JP Thompson SG Deeks JJ Altman DG . Measuring inconsistency in meta-analyses. BMJ. (2003) 327:557–60. doi: 10.1136/bmj.327.7414.557. PMID: 12958120 PMC192859

[20] Gu H Zhu T Ding J Yang Z Lu Y Guo G . The association between sarcopenia and clinical outcomes among Chinese patients with triple-negative breast cancer: a retrospective study. Front Oncol. (2025) 15:1402300. doi: 10.3389/fonc.2025.1402300. PMID: 39980560 PMC11839753

[21] Guo XY Wen RL Yu LF Lin H . Tumor size, HER-2 status, CA125, CEA, SII, and PNI: key predictors of pathological complete response in LABC patients. Am J Cancer Res. (2024) 14:4880–95. doi: 10.62347/yawk6271. PMID: 39553222 PMC11560830

[22] Hu J Dong J Yang X Ye Z Hu G . Erythrocyte modified controlling nutritional status as a biomarker for predicting poor prognosis in post-surgery breast cancer patients. Sci Rep. (2025) 15:2071. doi: 10.1038/s41598-024-83729-1. PMID: 39814814 PMC11736028

[23] Hua X Long ZQ Huang X Deng JP He ZY Guo L . The value of prognostic nutritional index (PNI) in predicting survival and guiding radiotherapy of patients with T1-2N1 breast cancer. Front Oncol. (2020) 9:1562. doi: 10.3389/fonc.2019.01562. PMID: 32083015 PMC7002465

[24] Hutajulu SH Astari YK Ucche M Kertia N Subronto YW Paramita DK . Prognostic significance of C-reactive protein (CRP) and albumin-based biomarker in patients with breast cancer receiving chemotherapy. PeerJ. (2025) 13:e19319. doi: 10.7717/peerj.19319. PMID: 40416620 PMC12103165

[25] Li W Li M Wang T Ma GZ Deng YF Pu D . Controlling nutritional status (CONUT) score is a prognostic factor in patients with resected breast cancer. Sci Rep. (2020) 10:6633. doi: 10.1038/s41598-020-63610-7. PMID: 32313183 PMC7171067

[26] Li X Cheng X Han Y Liu X Fang Y Ren S . PNI as a predictive biomarker: a novel nomogram of immunotherapy efficacy in advanced breast cancer. Front Oncol. (2025) 15:1534545. doi: 10.3389/fonc.2025.1534545. PMID: 40896424 PMC12395289

[27] Li Y Zhang Y Zhou Z Shang L Huang Y Lu X . Predictive value of controlling nutritional status score in postoperative recurrence and metastasis of breast cancer patients with HER2-low expression. Front Oncol. (2023) 13:1116631. doi: 10.3389/fonc.2023.1116631. PMID: 37492470 PMC10365291

[28] Mohri T Mohri Y Shigemori T Takeuchi K Itoh Y Kato T . Impact of prognostic nutritional index on long-term outcomes in patients with breast cancer. World J Surg Oncol. (2016) 14:170. doi: 10.1186/s12957-016-0920-7. PMID: 27349744 PMC4924248

[29] Oba T Maeno K Takekoshi D Ono M Ito T Kanai T . Neoadjuvant chemotherapy-induced decrease of prognostic nutrition index predicts poor prognosis in patients with breast cancer. BMC Cancer. (2020) 20:160. doi: 10.1186/s12885-020-6647-4. PMID: 32106833 PMC7045374

[30] Qiu Y Chen Y Shen H Yan S Li J Wu W . Naples prognostic score: a novel predictor of survival in patients with triple-negative breast cancer. J Inflammation Res. (2024) 17:5253–69. doi: 10.2147/JIR.S472917. PMID: 39135978 PMC11318610

[31] Sun L Liu JJ Wang D . Prognostic value of the preoperative prognostic nutritional index and systemic immuno-inflammatory index in Chinese breast cancer patients: a clinical retrospective cohort study. J Surg Oncol. (2023) 127:921–8. doi: 10.1002/jso.27210. PMID: 36734983

[32] Wang S Song Y Ding J Li M Wang Y Bai Y . Development and validation of a new immune-inflammatory-nutritional score to predict pathological complete response in triple-negative breast cancer undergoing neoadjuvant chemotherapy: a two-center study. J Inflammation Res. (2025) 18:9365–78. doi: 10.2147/JIR.S526429. PMID: 40687145 PMC12276740

[33] Wang Y Gao WX Wang S Zhang JJ Zhuang JR Wu YB . Significance of the inflammatory-immune-nutritional (IINS) score on postoperative survival and recurrence in breast cancer patients: a retrospective study. PeerJ. (2025) 13:e19950. doi: 10.7717/peerj.19950. PMID: 40860681 PMC12377354

[34] Xu T Zhang SM Wu HM Wen XM Qiu DQ Yang YY . Prognostic significance of prognostic nutritional index and systemic immune-inflammation index in patients after curative breast cancer resection: a retrospective cohort study. BMC Cancer. (2022) 22:1128. doi: 10.1186/s12885-022-10218-x. PMID: 36329394 PMC9632068

[35] Yamamoto S Adachi S Wada T Narui K Kimura A Oshi M . The modified Glasgow prognostic score and prognostic nutritional index as prognostic markers in patients with metastatic breast cancer treated with eribulin. In Vivo. (2022) 36:1854–9. doi: 10.21873/invivo.12903. PMID: 35738631 PMC9301441

[36] Yamanouchi K Murakami S Sato A Ogawa S Shinagawa H Kamohara Y . Integrated evaluation of inflammatory, nutritional, and sarcopenia markers to predict survival in metastatic breast cancer patients. In Vivo. (2023) 37:811–7. doi: 10.21873/invivo.13146. PMID: 36881066 PMC10026678

[37] Yang Z Zhang B Hou L Xie Y Cao X . Pre-operative prognostic nutritional index predicts the outcomes for triple-negative breast cancer. Tumour Biol. (2014) 35:12165–71. doi: 10.1007/s13277-014-2524-6. PMID: 25172099

[38] Zhu M Chen L Kong X Wang X Ren Y Liu Q . Controlling nutritional status (CONUT) as a novel postoperative prognostic marker in breast cancer patients: a retrospective study. BioMed Res Int. (2022) 2022:3254581. doi: 10.1155/2022/3254581. PMID: 36531650 PMC9757942

[39] Arici MO Kivrak Salim D Kocer M Alparslan AS Karakas BR Ozturk B . Predictive and prognostic value of inflammatory and nutritional indexes in patients with breast cancer receiving neoadjuvant chemotherapy. Med (Kaunas). (2024) 60:1849. doi: 10.3390/medicina60111849. PMID: 39597034 PMC11596226

[40] Buyuksimsek M Ogul A Mirili C Paydas S . Inflammatory markers predicting pathological complete response in cases with breast cancer treated by neoadjuvant chemotherapy. Eur J Breast Health. (2020) 16:229–34. doi: 10.5152/ejbh.2020.5556. PMID: 33062961 PMC7535993

[41] Onder T Oner I Karacin C Ates O . Valuable predictive power of prognostic nutritional index in metastatic breast cancer patients treated with CDK4/6 inhibitors. Jpn J Clin Oncol. (2025) 55:578–87. doi: 10.1093/jjco/hyaf036. PMID: 39997162

[42] Yildirim S Dogan A Akdag G Yasar ZY Bal H Kinikoglu O . The role of laboratory indices on treatment response and survival in breast cancer receiving neoadjuvant chemotherapy. Sci Rep. (2024) 14:12123. doi: 10.1038/s41598-024-63096-7. PMID: 38802494 PMC11130235

[43] Birsin Z Nazli I Alkan O Bükün HO Günaltili M Çerme E . Inflammatory and nutritional markers predicting pathological complete response to neoadjuvant therapy in HER2-positive breast cancer: a multicenter real-world study. J Clin Med. (2025) 14:7271. doi: 10.3390/jcm14207271. PMID: 41156142 PMC12565550

[44] Qu F Luo Y Peng Y Yu H Sun L Liu S . Construction and validation of a prognostic nutritional index-based nomogram for predicting pathological complete response in breast cancer: a two-center study of 1,170 patients. Front Immunol. (2023) 14:1335546. doi: 10.3389/fimmu.2023.1335546. PMID: 38274836 PMC10808698

[45] Zhang XW Ge YZ Song MM Ruan GT Xie HL Hu CL . Prognostic power of nutrition-inflammation indicators in patients with breast cancer. Clin Breast Cancer. (2023) 23:e312–21. doi: 10.1016/j.clbc.2023.04.009. PMID: 37236827

[46] Peng P Chen L Shen Q Xu Z Ding X . Prognostic nutritional index (PNI) and controlling nutritional status (CONUT) score for predicting outcomes of breast cancer: a systematic review and meta-analysis. Pak J Med Sci. (2023) 39:1535–41. doi: 10.12669/pjms.39.5.7781. PMID: 37680798 PMC10480717

[47] Kim Y Lee JH Cho ES Lee HS Shin SJ Park EJ . Albumin-myosteatosis gauge as a novel prognostic risk factor in patients with non-metastatic colorectal cancer. J Cachexia Sarcopenia Muscle. (2023) 14:860–8. doi: 10.1002/jcsm.13183. PMID: 36696881 PMC10067505

[48] Hoogenboezem EN Duvall CL . Harnessing albumin as a carrier for cancer therapies. Adv Drug Delivery Rev. (2018) 130:73–89. doi: 10.1016/j.addr.2018.07.011. PMID: 30012492 PMC6200408

[49] Li YT Zhou XS Han XM Tian J Qin Y Zhang T . Pretreatment serum albumin-to-alkaline phosphatase ratio is an independent prognosticator of survival in patients with metastatic gastric cancer. World J Gastrointest Oncol. (2022) 14:1002–13. doi: 10.4251/wjgo.v14.i5.1002. PMID: 35646278 PMC9124991

[50] Duran-Güell M Flores-Costa R Casulleras M López-Vicario C Titos E Díaz A . Albumin protects the liver from tumor necrosis factor α-induced immunopathology. FASEB J. (2021) 35:e21365. doi: 10.1096/fj.202001615RRR. PMID: 33496031

[51] Xiang M Zhang H Tian J Yuan Y Xu Z Chen J . Low serum albumin levels and high neutrophil counts are predictive of a poorer prognosis in patients with metastatic breast cancer. Oncol Lett. (2022) 24:432. doi: 10.3892/ol.2022.13552. PMID: 36311691 PMC9608081

[52] Xu Y Qi Y Lu Z Tan Y Chen D Luo H . Navigating precision: the crucial role of next-generation sequencing recurrence risk assessment in tailoring adjuvant therapy for hormone receptor-positive, human epidermal growth factor receptor 2-negative early breast cancer. Cancer Biol Ther. (2024) 25:2405060. doi: 10.1080/15384047.2024.2405060. PMID: 39304993 PMC11418226

[53] He Y Zhai A Qin K Zhou X Yu Y Zhang Z . Assessment of the efficacy and safety of anlotinib for the treatment of recurrent epithelial ovarian cancer. Discov Oncol. (2024) 15:383. doi: 10.1007/s12672-024-01267-8. PMID: 39207632 PMC11362443

[54] Park J Hsueh PC Li Z Ho PC . Microenvironment-driven metabolic adaptations guiding CD8(+) T cell anti-tumor immunity. Immunity. (2023) 56:32–42. doi: 10.1016/j.immuni.2022.12.008. PMID: 36630916

[55] Guo M Liu MYR Brooks DG . Regulation and impact of tumor-specific CD4(+) T cells in cancer and immunotherapy. Trends Immunol. (2024) 45:303–13. doi: 10.1016/j.it.2024.02.005. PMID: 38508931

[56] Laumont CM Nelson BH . B cells in the tumor microenvironment: Multi-faceted organizers, regulators, and effectors of anti-tumor immunity. Cancer Cell. (2023) 41:466–89. doi: 10.1016/j.ccell.2023.02.017. PMID: 36917951

[57] Virassamy B Caramia F Savas P Sant S Wang J Christo SN . Intratumoral CD8(+) T cells with a tissue-resident memory phenotype mediate local immunity and immune checkpoint responses in breast cancer. Cancer Cell. (2023) 41:585–601.e8. doi: 10.1016/j.ccell.2023.01.004. PMID: 36827978

[58] King RJ Singh PK Mehla K . The cholesterol pathway: impact on immunity and cancer. Trends Immunol. (2022) 43:78–92. doi: 10.1016/j.it.2021.11.007. PMID: 34942082 PMC8812650

