# Supplementary figures and images for: Predicting the prognosis of breast cancer patients by using nutrition-based index: a systematic review and meta-analysis
Source: Front Oncol. 2026 May 11;16:1775719. doi: 10.3389/fonc.2026.1775719 (PMC13198998; doi:10.3389/fonc.2026.1775719)

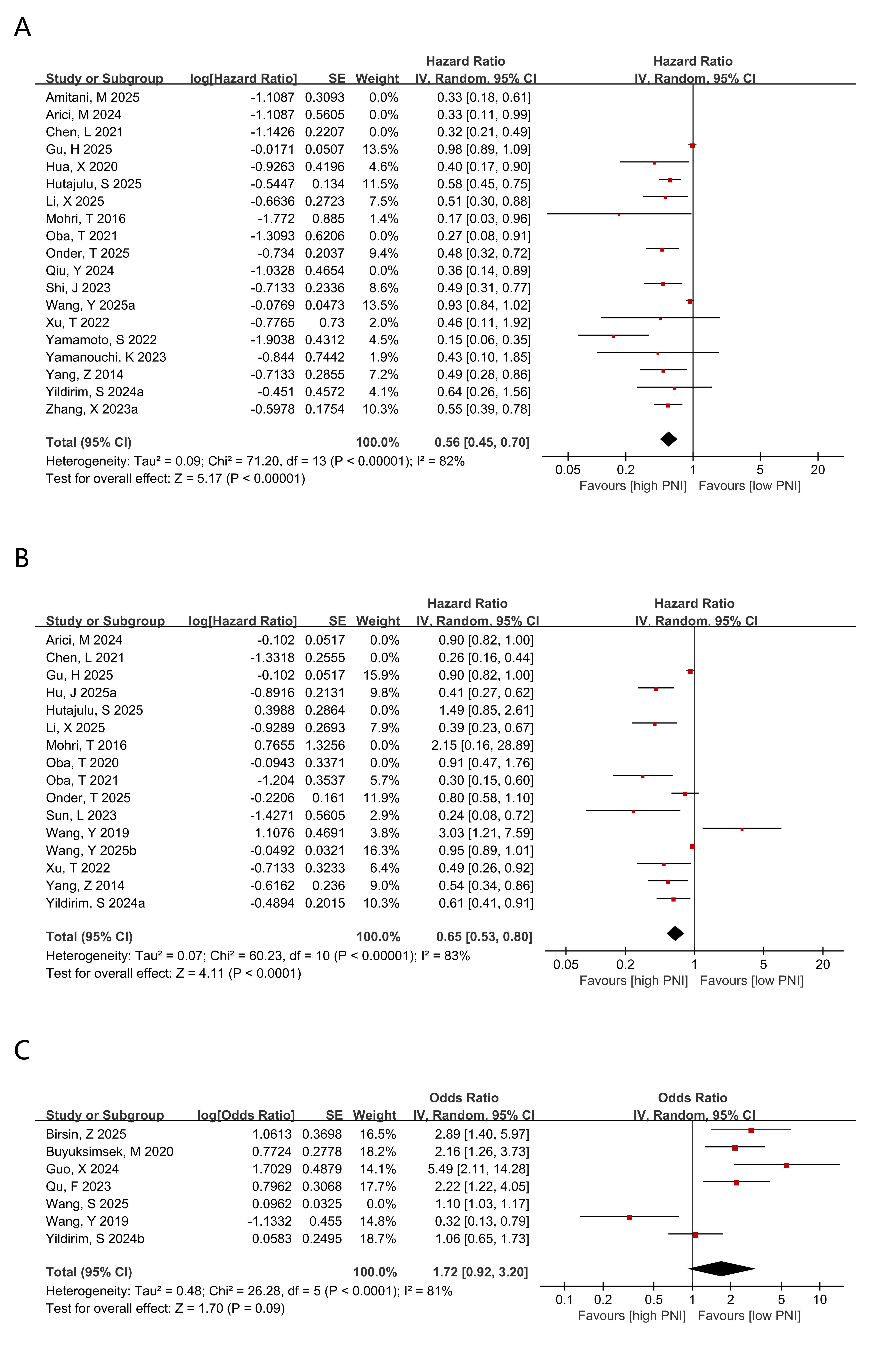

Supplement: Supplementary file 1 [file Image1.jpeg]

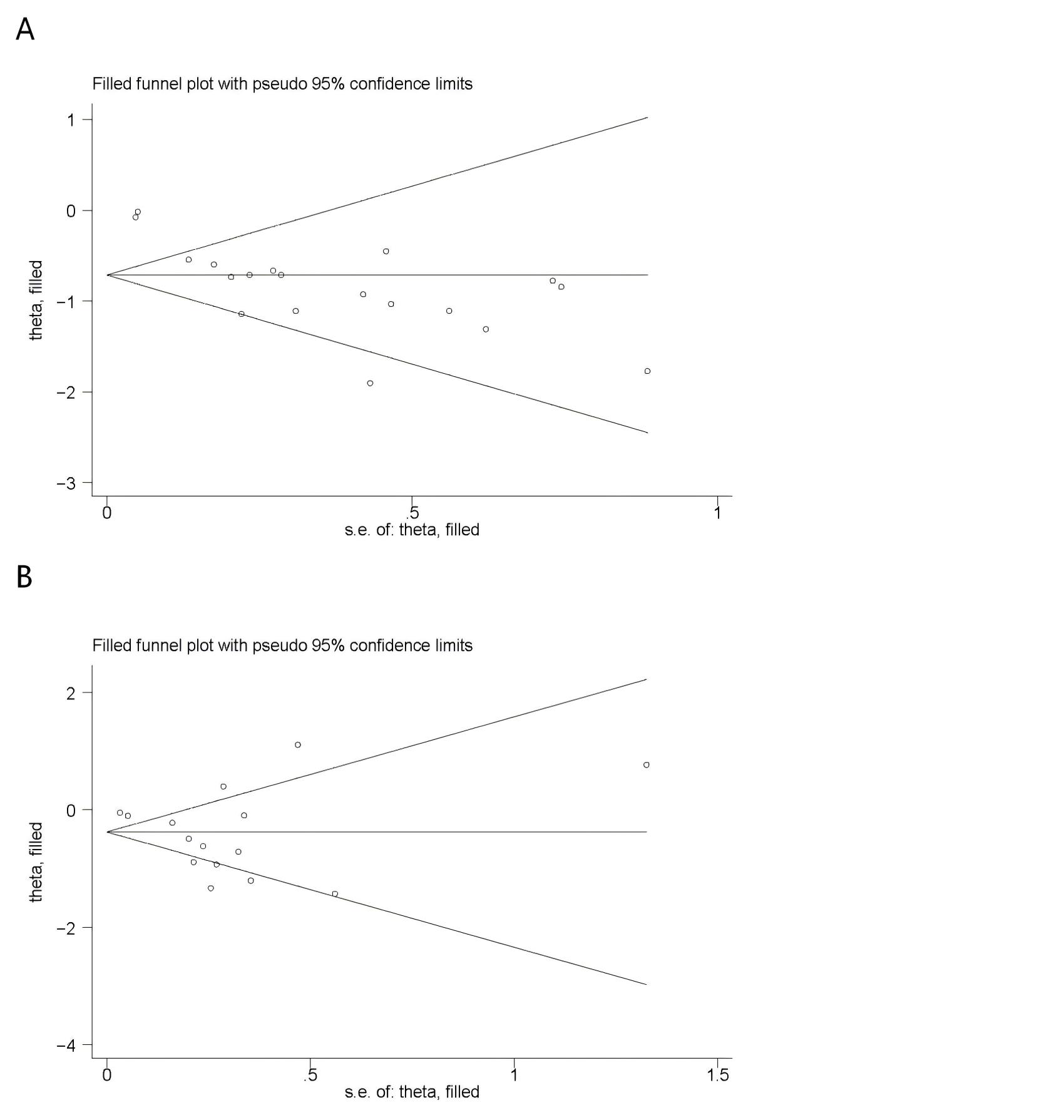

Supplement: Supplementary file 2 [file Image2.jpeg]
