# Supplementary material for: Predicting the prognosis of breast cancer patients by using nutrition-based index: a systematic review and meta-analysis
Source: Front Oncol. 2026 May 11;16:1775719. doi: 10.3389/fonc.2026.1775719 (PMC13198998; doi:10.3389/fonc.2026.1775719)
Supplement: Supplementary file 3 [file Table1.docx]

| Supplementary Table S1. Detailed search strategy in four databases. | |
| --- | --- |
| Database | Search strategy |
| Pubmed | (((Prognostic Nutritional Index) OR (PNI)) OR (((Controlling nutritional status) OR (CONUT score)) OR (CONUT))) AND (("Breast Neoplasms"[Mesh]) OR ((((((((((((((((((((Breast Neoplasm) OR (Breast Tumors)) OR (Breast Tumor)) OR (Breast Cancer)) OR (Cancer of Breast)) OR (Cancer of the Breast)) OR (Malignant Neoplasm of Breast)) OR (Breast Malignant Neoplasm)) OR (Breast Malignant Neoplasms)) OR (Malignant Tumor of Breast)) OR (Breast Malignant Tumor)) OR (Breast Malignant Tumors)) OR (Mammary Cancer)) OR (Mammary Cancers)) OR (Human Mammary Neoplasm)) OR (Human Mammary Neoplasms)) OR (Breast Carcinoma)) OR (Breast Carcinomas)) OR (Human Mammary Carcinomas)) OR (Human Mammary Carcinoma))) |
| Embase | ((Prognostic Nutritional Index or PNI or (Controlling nutritional status or CONUT score or CONUT)) and (Breast Neoplasms or (Breast Neoplasm or Breast Tumors or Breast Tumor or Breast Cancer or Cancer of Breast or Cancer of the Breast or Malignant Neoplasm of Breast or Breast Malignant Neoplasm or Breast Malignant Neoplasms or Malignant Tumor of Breast or Breast Malignant Tumor or Breast Malignant Tumors or Mammary Cancer or Mammary Cancers or Human Mammary Neoplasm or Human Mammary Neoplasms or Breast Carcinoma or Breast Carcinomas or Human Mammary Carcinomas or Human Mammary Carcinoma))).af. |
| Web of Science | (((Prognostic Nutritional Index) OR (PNI)) OR (((Controlling nutritional status) OR (CONUT score)) OR (CONUT))) AND ((Breast Neoplasms) OR ((((((((((((((((((((Breast Neoplasm) OR (Breast Tumors)) OR (Breast Tumor)) OR (Breast Cancer)) OR (Cancer of Breast)) OR (Cancer of the Breast)) OR (Malignant Neoplasm of Breast)) OR (Breast Malignant Neoplasm)) OR (Breast Malignant Neoplasms)) OR (Malignant Tumor of Breast)) OR (Breast Malignant Tumor)) OR (Breast Malignant Tumors)) OR (Mammary Cancer)) OR (Mammary Cancers)) OR (Human Mammary Neoplasm)) OR (Human Mammary Neoplasms)) OR (Breast Carcinoma)) OR (Breast Carcinomas)) OR (Human Mammary Carcinomas)) OR (Human Mammary Carcinoma))) (Topic) |
| Chochrane | ((Prognostic Nutritional Index or PNI or (Controlling nutritional status or CONUT score or CONUT)) and (Breast Neoplasms or (Breast Neoplasm or Breast Tumors or Breast Tumor or Breast Cancer or Cancer of Breast or Cancer of the Breast or Malignant Neoplasm of Breast or Breast Malignant Neoplasm or Breast Malignant Neoplasms or Malignant Tumor of Breast or Breast Malignant Tumor or Breast Malignant Tumors or Mammary Cancer or Mammary Cancers or Human Mammary Neoplasm or Human Mammary Neoplasms or Breast Carcinoma or Breast Carcinomas or Human Mammary Carcinomas or Human Mammary Carcinoma))).af. |
