# Supplementary material for: Predicting the prognosis of breast cancer patients by using nutrition-based index: a systematic review and meta-analysis
Source: Front Oncol. 2026 May 11;16:1775719. doi: 10.3389/fonc.2026.1775719 (PMC13198998; doi:10.3389/fonc.2026.1775719)
Supplement: Supplementary file 4 [file Table2.docx]

| Supplementary Table S2. Quality evaluation of the eligible studies with Newcastle–Ottawa scale. | | | | | | | | | |
| --- | --- | --- | --- | --- | --- | --- | --- | --- | --- |
| Study | Selection | | | | Comparability | | Outcome | | |
|  | Representative-ness | Selection of  non-exposed | Ascertainment  of exposure | Outcome not present at start | Comparability on most important factors | Comparability on other risk factors | Assessment of outcome | Long enough follow-up (median≥1 year) | Adequacy  (completeness) of follow-up |
| Gu, H et al. | * | * | * | * | - | - | * | * | * |
| Shi, J et al. | * | - | * | * | - | - | * | * | * |
| Qu, F et al. | * | * | * | * | * | - | * | * | * |
| Zhu, M et al. | * | * | * | * | - | - | * | * | * |
| Li, W et al. | * | * | * | * | - | - | * | * | * |
| Wang, S et al. | * | * | * | * | - | - | * | * | * |
| Hu, J et al. | * | * | * | * | - | - | * | * | * |
| Mohri, T et al. | * | * | * | * | - | * | * | * | * |
| Birsin, Z et al. | * | * | * | * | - | - | * | * | * |
| Buyuksimsek, M et al. | * | * | * | * | - | * | * | * | * |
| Yamanouchi, K et al. | * | * | * | * | - | - | * | * | * |
| Yamamoto, S et al. | * | * | * | * | - | - | * | * | * |
| Qiu, Y et al. | * | * | * | * | - | - | * | * | * |
| Oba, T et al. | * | * | * | * | - | - | * | * | * |
| Li, X et al. | * | * | * | * | - | - | * | * | * |
| Arici, M et al. | * | * | * | * | - | - | * | * | * |
| Wang, Y et al. | * | * | * | * | * | * | * | * | * |
| Li, Y et al. | * | * | * | * | - | - | * | * | * |
| Yang, Z et al. | * | * | * | * | - | - | * | * | * |
| Chen, L et al. | * | * | * | * | - | - | * | * | * |
| Oba, T et al. | * | * | * | * | * | - | * | * | * |
| Zhang, X et al. | * | - | * | * | - | - | * | * | * |
| Amitani, M et al. | * | * | * | * | * | * | * | * | * |
| Hutajulu, S et al. | * | * | * | * | - | * | * | * | * |
| Xu, T et al. | * | * | * | * | - | - | * | * | * |
| Huang, Z et al. | * | * | * | * | - | * | * | * | * |
| Sun, L et al. | * | * | * | * | - | - | * | * | * |
| Yildirim, S et al. | * | * | * | * | - | - | * | * | * |
| Wang, Y et al. | * | * | * | * | - | - | * | * | * |
| Guo, X et al. | * | * | * | * | - | - | * | * | * |
| Onder, T et al. | * | * | * | * | - | - | * | * | * |
| Hua, X et al. | * | * | * | * | - | - | * | * | * |
| *indicates criterion met; - indicates significant of criterion not met. | | | | | | | | | |
