# Supplementary material for: Predicting the prognosis of breast cancer patients by using nutrition-based index: a systematic review and meta-analysis
Source: Front Oncol. 2026 May 11;16:1775719. doi: 10.3389/fonc.2026.1775719 (PMC13198998; doi:10.3389/fonc.2026.1775719)
Supplement: Supplementary file 5 [file Table3.docx]

| Potentially overlapping reports | region | Recruitment period | Population / subtype | Sample size | Key distinguishing feature | Overlap conclusion |
| --- | --- | --- | --- | --- | --- | --- |
| Hu J 2025a vs Hu J 2025b | China | 2012-2018 / 2012-2018 | BC / BC | 536 / 536 | Different nutritional index: PNI  vs CONUT; both reported DFS | Same underlying cohort,  different index reports |
| Zhang X 2023a vs Zhang X 2023b | Multicenter | 2012-2019 / 2012-2019 | BC / BC | 1151 / 1151 | Different nutritional index: PNI  vs CONUT; both reported OS | Same underlying cohort,  different index reports |
| Li Y 2023a vs Li Y 2023b | China | 2014-2017 / 2014-2017 | HER2-low BC /  HER2-positive BC | 697 / 202 | Different biologic subtypes | Independent patient cohorts |
| Wang Y 2025a vs Wang Y 2025b | China | 2017-2022 / 2017-2022 | BC / BC | 200 / 200 | Same index (PNI), different reported endpoints:  Wang Y 2025a OS/DFS; Wang Y 2025b pCR | Same underlying cohort,  different endpoint reports |
| Yildirim S 2024a vs Yildirim S 2024b | Turkey | 2010-2022 / 2010-2022 | BC / BC | 624 / 624 | Same index (PNI), different reported endpoints:  Yildirim S 2024a OS/DFS; Yildirim S 2024b pCR | Same underlying cohort,  different endpoint reports |
