# Supplementary material for: Predicting the prognosis of breast cancer patients by using nutrition-based index: a systematic review and meta-analysis
Source: Front Oncol. 2026 May 11;16:1775719. doi: 10.3389/fonc.2026.1775719 (PMC13198998; doi:10.3389/fonc.2026.1775719)
Supplement: Supplementary file 6 [file Table4.docx]

| Author | study period | region | Center / hospital | Population | Nutritional indices | Treatment method | Overlap assessment |
| --- | --- | --- | --- | --- | --- | --- | --- |
| Chen, L 2021 | 1998-2016 | China | Cancer Hospital Chinese Academy of Medical Sciences | BC | 51.0（PNI） | NACT and surgery | Different nutritional index: Chen, L 2021 PNI  vs Zhu, M 2022 CONUT |
| Zhu, M 2022 | 2010-2016 | China | Cancer Hospital Chinese Academy of Medical Sciences | BC | 1.0（CONUT） | Surgery |  |
| Hua, X 2020 | 2010-2012 | China | Sun Yat-sen University Cancer Center (SYSUCC) | BC | 52.0（PNI） | Surgery | Different nutritional index: Hua, X 2020 PNI  vs Huang, Z 2020 CONUT |
| Huang, Z 2020 | 2010-2019 | China | Sun Yat-sen University Cancer Center (SYSUCC) | BC | 3.0（CONUT） | Surgery |  |
| Gu, H 2025 | 2012-2023 | China | First Affiliated Hospital of Wenzhou Medical University | TNBC | 51.0（PNI） | Surgery | No apparent overlap |
| Guo, X 2024 | 2010-2021 | China | Affiliated Fuzhou First Hospital of Fujian Medical University | BC | 47.0（PNI） | NACT and surgery | No apparent overlap |
| Li, W 2020 | 2007-2010 | China | West China Hospital of Sichuan University | BC | 3.0（CONUT） | Surgery | No apparent overlap |
| Li, X 2025 | 2022-2024 | China | First Affiliated Hospital of Zhengzhou University | Advanced breast cancer | 47.5（PNI） | Immunotherapy | No apparent overlap |
| Qiu, Y 2024 | 2016-2020 | China | The Affiliated Lihuili Hospital, Ningbo University | TNBC | 50.9（PNI） | Surgery | No apparent overlap |
| Sun, L 2023 | 2013-2020 | China | Second Affiliated Hospital of Jilin University | BC | 51.1(PNI) | Surgery | No apparent overlap |
| Wang, S 2025 | 2018-2023 | China | Xijing Hospital, Fourth Military Medical University | TNBC | 53.6（PNI） | NACT and surgery | No apparent overlap |
| Wang, Y 2019 | 2013-2018 | China | Renji Hospital, School of Medicine, Shanghai Jiao Tong University | Locally advanced breast cancer | 55.0（PNI） | NACT and surgery | No apparent overlap |
| Xu, T 2022 | 2013-2020 | China | Meizhou People's Hospital | BC | 53.0（PNI） | Surgery | No apparent overlap |
| Yang, Z 2014 | 2003-2013 | China | Tianjin Medical University Cancer Institute and Hospital | TNBC | 48.7（PNI） | Surgery | No apparent overlap |
