# Supplementary material for: Predicting the prognosis of breast cancer patients by using nutrition-based index: a systematic review and meta-analysis
Source: Front Oncol. 2026 May 11;16:1775719. doi: 10.3389/fonc.2026.1775719 (PMC13198998; doi:10.3389/fonc.2026.1775719)
Supplement: Supplementary file 7 [file Table5.docx]

| Variables | Coefficient | P value | 95% CI | R² | τ² |
| --- | --- | --- | --- | --- | --- |
| **Treatment method** |  |  |  |  |  |
| NACT and Surgery vs Surgery | -0.625 | 0.025 | [-1.173,-0.078] | 100% | 0 |
| Other vs Surgery | 0.263 | 0.425 | [-0.383,0.909] |  |  |
| **Adjustment** |  |  |  |  |  |
| Multivariate vs Univariate | 0.359 | 0.139 | [-0.117,0.835] |  |  |
| **Tumor stage** |  |  |  |  |  |
| Non-metastatic vs Metastatic | 1.007 | 0.007 | [0.270,1.743] |  |  |
| Mixed vs Metastatic | 0.298 | 0.128 | [-0.086,0.681] |  |  |
